# Convolutional Kernel-Embedded *E*(3)-Equivariant Networks (KEN): Overcoming Architectural Rigidity in Machine Learning Interatomic Potentials

**DOI:** 10.1021/acs.jctc.6c01109

**Published:** 2026-10-02

**Authors:** Jha Gautam

**Affiliations:** Hylleraas Centre for Quantum Molecular Sciences, Department of Chemistry, 6305 University of Oslo, Postboks 1033 Blindern, 0315 Oslo, Norway

## Abstract

Machine learning interatomic potentials (MLIPs) have emerged as scalable alternatives to first-principles methods such as density functional theory (DFT). Among them, *E*(3)-equivariant graph neural networks (GNNs) like PACE and MACE are highly accurate but structurally rigid, limiting the incorporation of rich, multichannel electronic descriptors. We introduce convolutional kernel-embedded *E*(3)-equivariant networks (KEN), a hybrid architecture that combines the local environmental extraction of 2D convolutional kernels with the many-body expressivity of an *E*(3)-equivariant GNN backbone. This design preserves symmetry while enabling flexible, multimodal input features without increasing training data requirements. Trained and tested on heavy element 26 (HE26) dataset, KEN was compared with MACE-osaka26 model. Further validation on lattice parameter prediction through Birch–Murnaghan equation of state, magnetic moment prediction, and comparison with CHGNet framework, and microcanonical molecular dynamics confirms robust energy conservation and accurate reproduction of the forces, and thus, demonstrates a viable pathway to overcoming architectural rigidity in state-of-the-art MLIPs.

## Graphical abstract

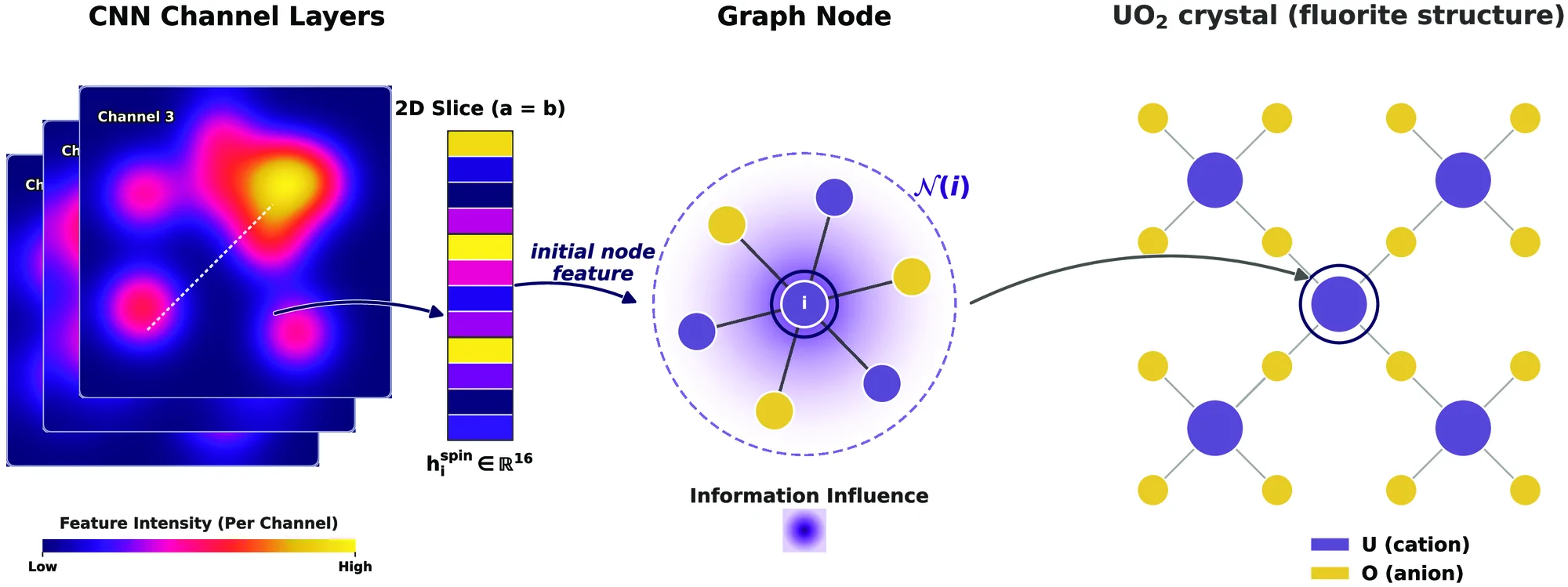

## 1 Introduction

The development of machine learning interatomic potentials (MLIPs) has revolutionized computational materials science by offering near-quantum mechanical accuracy at a computational cost scaling linearly with the number of atoms. In particular, the introduction of *E*(3)-equivariant graph neural networks (GNNs), such as NequIP,^1^ Allegro,^2^ and the Atomic Cluster Expansion (PACE)^3–5^ formalized through higher-order message-passing schemes like MACE,^6^ has established a new state-of-the-art for structural and thermodynamic modeling. By embedding the fundamental symmetries of Euclidean space directly into the tensor operations of the neural network graph, these frameworks shrinks the size of training data required for desired chemical accuracy.

Despite these breakthroughs, a critical research gap remains at the intersection of GNN based machine learning and strongly correlated electronic systems as such architectures construct potential energy surfaces using exclusively the 3D Cartesian coordinates and static chemical identities of the constituent nuclei.^7^ While this coordinate-only paradigm holds for simple metals and covalent semiconductors, it breaks down in strongly correlated materials where electronic, magnetic, or spin degrees of freedom can decouple from the underlying geometric framework. One such case is magnetic MLIPs,^8^ where current architecture is limited to explicit incorporation of magnetic information in either GNN,^6^ or Convolutional Neural Network (CNN)^9^ frameworks. MLIPs such as mMTP^10^ or mACE^11^ with explicit spin variables exist and have shown impressive results for magnetic systems but they require tailored datasets, constrained-spin sampling, active-learning workflows in spin space, and added complexity at training and sometimes at runtime.^12,13^ This limitation is particularly pronounced in actinides, where heavily localized 5f electrons drive complex crystal-field splittings, spin orbit coupling, variable valence states, and diverse magnetic moment configurations,^14–16^ for example, Alzate-Vargas et al. reported the importance of active learning for MLIP training of uranium nitride (UN).^17^ Nguyen et al.^18^ used GAP^19^ potentials to simulate actinide molten salt and found good theoretical and experimental agreement but they only investigated 1 oxidation state of Th and U whereas MLIPs for mixed valence molten salts is absent in literature. Hence, when a single geometric coordinates layout can correspond to multiple distinct electronic and spin states, it can render purely coordinate-based potentials effectively ill-posed, leading to inaccurate force predictions, nonphysical coordinate explosions, and structural instabilities during finite-temperature simulations.

To address this challenge, we introduce a multimodal interatomic potential architecture that couples *E*(3)-equivariant many-body graph convolutions with a two-dimensional (2D) convolutional edge feature extractor. Rather than relying solely on geometric coordinates, our framework explicitly ingests distance-screened electronic spin-interaction matrices. By applying a deterministic canonical ordering map upstream of grid construction, the network processes these matrices through 2D element-segregated convolutional kernels to capture localized magnetic neighborhood interactions into permutation-equivariant scalar embeddings. These electronic spin descriptors are injected directly into the invariant scalar channels of an *E*(3)-equivariant GNN backbone, establishing a flexible architecture that is jointly trained on total energies and conservative forces via the graph network, and on localized magnetic moments via the CNN-derived spin channels.

In this work, we demonstrate the predictive capabilities of this hybrid architecture by training and testing on HE26 database^20^ as it represents a magnetically and chemically diverse dataset of actinides. The compiled potential is compared to MACE level training and testing metrics, along with a comparison against CHGNet for magnetic moment prediction. KEN MLIPs (termed as KENJI) accurately reproduces experimental structural properties such as lattice parameters by near-equilibrium bulk equation of state, demonstrates robust energy conservation with low drift under microcanonical (NVE) dynamics, and delivers long range stochastic NVT molecular dynamics stability.

## 2 Methodology and Architecture

The architectural workflow couples a 2D spatial grid-based convolutional framework, handling localized electronic spin correlations through a Heisenberg spin model,^21^ with a 3D GNN operating under strict Euclidean space equivariance (*E*(3)) (Figure 1).

**Figure 1:**
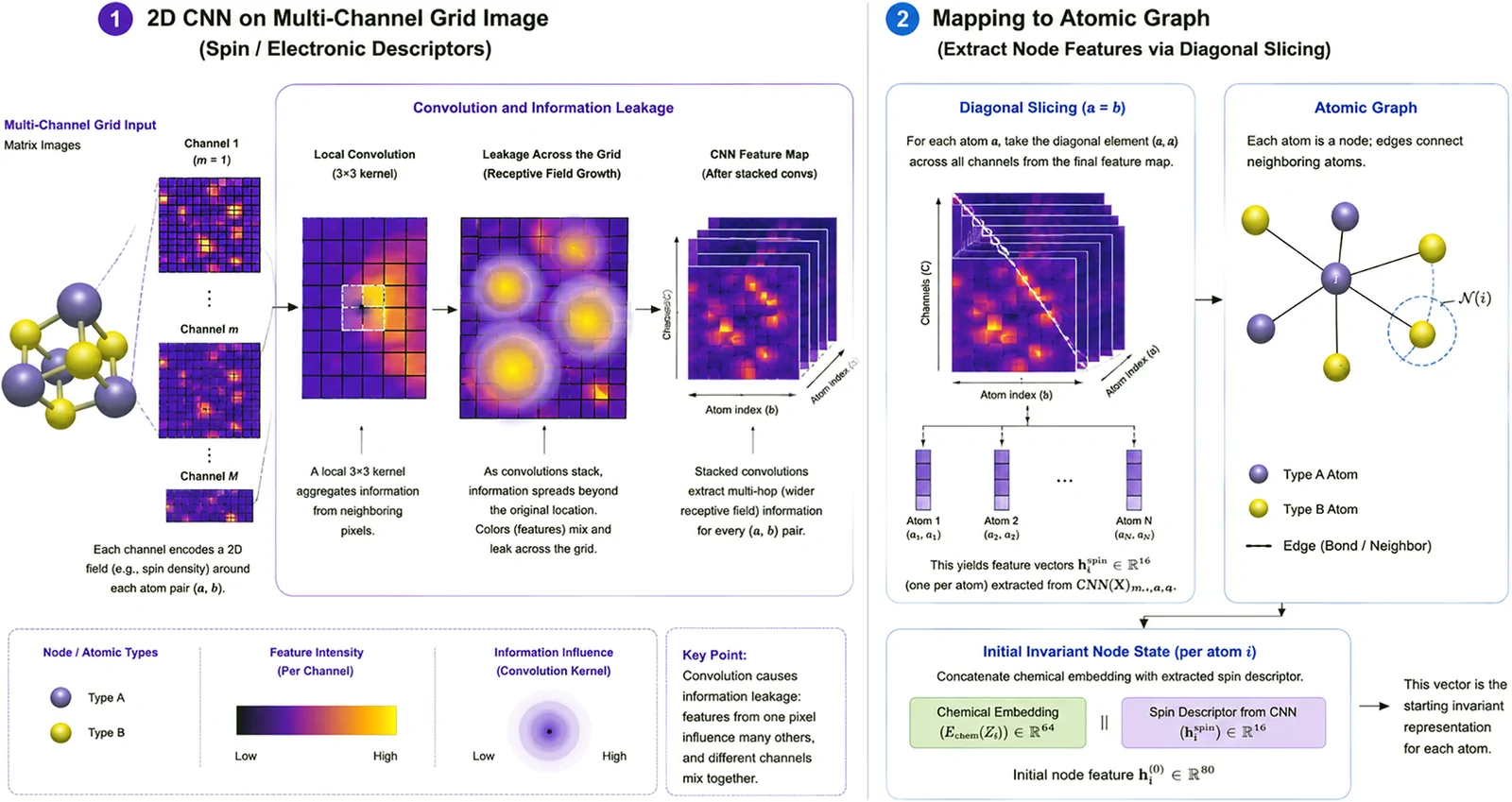
KEN architecture diagram for embedding 2D CNN with atomic graphs.

### 2.1 2D Spin Interaction Descriptor Layer

To move beyond coordinate-only constraints, we incorporate localized electronic spin degrees of freedom by passing stacked physical interaction matrices $X\in R^{B\times C\times N_{\max}\times N_{\max}}$ through a 2D CNN Feature Extractor ( $M_{\mathrm{CNN}}$ ). Here, *B* denotes the batch size, *C* = 3 represents distinct, normalized magnetic interaction channels (spin-pair interactions, spin-distance decay, and atomic self-interaction diagonals: Section 2.2.1), and *N*_max_ is the maximum structural size parameter within the batched configuration.

The multichannel grids are processed through successive convolutional operations, using 3 × 3 kernels with unit padding to preserve the spatial grid dimension while aggregating information from each atom pair’s local neighborhood on the interaction grid

1 $$H_{\mathrm{grid}}={\mathrm{Conv}}_{1\times 1}\left(\sigma \left({\mathrm{BN}}_{2}\left({\mathrm{Conv}}_{3\times 3}^{\left(2\right)}\left(\sigma \left({\mathrm{BN}}_{1}\left({\mathrm{Conv}}_{3\times 3}^{\left(1\right)}\left(X\right)\right)\right)\right)\right)\right)\right)$$

where BN_*n*_ represents batch normalization layers, and σ denotes the Sigmoid Linear Unit (SiLU) activation function. The 3 × 3 convolutions ensure that the network extracts multihop information regarding how a specific atom is embedded within its local magnetic and topological environment. The localized features are subsequently mapped from the dense 2D grid into the node space of the structural graph via a diagonal slicing operator (**M**_map_)­

2 $$h_{i}^{\mathrm{spin}}=H_{\mathrm{grid}}\left[m,:,a,a\right]\in R^{16}$$

where *m* represents the global molecular index within the batch, and *a* represents the localized, intra-molecular atomic pointer evaluated from the configuration layout. This operation extracts 16 scalar attributes per node that function as invariant (0*e*) geometric inputs for the downstream equivariant message-passing layers.

### 2.2 Canonical Ordering Hypothesis for the Magnetic Branch

#### 2.2.1 Multi-Channel Spin Interaction Tensor

For each molecular structure containing *N* atoms, localized electronic degrees of freedom are mapped into a multichannel two-dimensional interaction tensor space $T\in R^{3\times N\times N}$ through three distinct nonlinear physical mapping operators (eqs 3–6):

**Pairwise spin configurations (Channel 0)** encode long-range interatomic magnetic alignment or anti-alignment tendencies bounded across a hyperbolic tangent envelope:

3 $$S_{\mathrm{ij}}=\tanh\left(\mu_{i}\mu_{j}\right)$$

4 $$T_{0,\mathrm{ij}}=\tanh\left(\frac{S_{\mathrm{ij}}}{\sigma \left(S\right)\;+\;\text{ϵ}}\right)$$

where σ(·) denotes the standard deviation evaluated dynamically over the structural matrix slice and ϵ = 10^–8^ is a smoothing stabilizer to ensure numerical safety during zero-variance states.

**Distance-screened exchange layouts (Channel 1)** exponentially attenuate the pre-enveloped spin-coupling magnitudes as a function of the minimum-image periodic interatomic distances *r*_*ij*_, mimicking the localized decay of short-range electronic exchange interactions in bulk environments:

5 $$T_{1,\mathrm{ij}}=\tanh\left(\frac{S_{\mathrm{ij}}\cdot \exp\left(-r_{\mathrm{ij}}\right)}{\sigma \left(S\cdot \exp\left(-r\right)\right)\;+\;\text{ϵ}}\right)$$

**Atomic reference diagonal states (Channel 2)** isolate self-interaction terms on the grid diagonal to anchor isolated elemental initial spin parameters:

6 $$T_{2,\mathrm{ii}}=\tanh\left(\frac{\mu_{i}}{\sigma \left(\mu_{\mathrm{diag}}\right)\;+\;\text{ϵ}}\right),,T_{2,\mathrm{ij}}=0,\left(i\neq j\right)$$

To form rigid batches suitable for parallel graphics acceleration, individual channel layouts are zero-padded up to a uniform spatial canvas matching the largest system size present in the current mini-batch, yielding the continuous tensor $X_{\mathrm{grid}}\in R^{B\times 3\times N_{\max}\times N_{\max}}$ .

##### Canonical Ordering

Because Conv_3×3_ mixes spatial neighbors, it does not commute with an arbitrary permutation matrix **P**. In order to maintain equivariance, every input structure, regardless of its native atom-listing order, is passed through a deterministic canonicalization map C(·) before grid construction, which is implemented as a three-key lexicographic sort:

1. Primary key—atomic species *Z*_*i*_: grouping atoms by element keeps the CNN’s channel semantics chemically consistent across the dataset.
2. Secondary key—radial distance from the structure’s centroid *d*_*i*_ = ∥**r**_*i*_ – **r̅**∥, computed from positions wrapped into the primary periodic cell to resolve minimum-image ambiguity. This key is rotation-invariant and carries no sign ambiguity, and maintain the equivariance up to machine precision.
3. Tertiary key—original array index *i*: serves as a deterministic fallback to guarantee a stable total order within the sorting algorithm. While geometric relaxation tolerances ensure that no two atoms of the same species share an identical centroid distance in practice (∥*d*_*i*_ – *d*_*j*_∥ ≫ δ_mach_), this key is used strictly as an arbitrary implementation tie-breaker for algorithmic stability in software.

Formally, letting $Z\in N^{N}$ and $d\in R^{N}$ denote the per-atom species and centroid distances, the canonicalization map is given by

7 C(structure)=lexsort(Z,d,i)

where **Z** is the primary sort key, **d** is the secondary (tie-breaking) key, and **i** is the tertiary key.

##### Lemma 1 (Permutation Equivariance and Canonical Invariance of Spin Interaction Channels)

Let a molecular structure within a batched system be defined by an arbitrary atomic indexing sequence {1, 2, ..., *N*}. Let **P** ∈ {0, 1}^*N*×*N*^ be an arbitrary permutation matrix representing a reordering of the atomic indices, where **P** ^T^ **P** = **I**. Under atomic reindexing, the 1D atomic spin vector transforms as **μ**′ = **Pμ** and the interatomic 3D distances transform as **R**′_dist_ = **PR**_dist_ **P** ^T^. Because the physical channel operators (eqs 3–6) depend strictly on pairwise quantities (μ_*i*_, μ_*j*_) and invariant interatomic distances *r*_*ij*_ = *r*_π(*i*)π(*j*)_, and because standard deviation σ(·) is a symmetric function over multisets invariant to element reordering, each individual channel transforms strictly matrix-equivariantly:

8 $$T_{c}^{\prime }\left[m,:,:\right]={\mathrm{PT}}_{c}\left[m,:,:\right]P^{T},\forall c\in \left\{0,1,2\right\}$$

Stacking these channels forms the multichannel interaction tensor **X**_grid_, which naturally inherits exact slicewise matrix permutation equivariance: **X**_grid_^′^[*m*, :, :, :] = **PX**_grid_[*m*, :, :, :]**P** ^T^. Furthermore, passing this equivariant grid through the canonical ordering map C(structure) resolves the permutation dependency entirely, mapping the representation into a strictly invariant state. In practical, physically relaxed molecular structures, geometric asymmetry and structural optimization tolerances dictate that no two atoms of the same species share an identical centroid distance down to machine precision (∥*d*_*i*_ – *d*_*j*_∥ ≫ δ_mach_ ≈ 10^–16^). Because the secondary radial key forms a strict total order across all valid chemical configurations, the tertiary fallback key remains dormant. Consequently, the mapped canonical tensor is exactly invariant to the raw input listing sequence up to floating-point precision:

9 $$X_{\mathrm{grid}}^{\mathrm{can}}=M_{\mathrm{grid}}\left(C\left(P\cdot \mathrm{structure}\right)\right)=M_{\mathrm{grid}}\left(C\left(\mathrm{structure}\right)\right),\forall P$$

#### 2.2.2 Permutation Equivariance for Spatial Convolutions via Canonicalization

For spatial operations such as 3 × 3 convolutions, neighbor-mixing kernels require a deterministic spatial frame. To achieve this, the input structure under an arbitrary raw indexing sequence π (represented by a permutation matrix **P** ∈ {0, 1}^*N*×*N*^) is first passed through the canonicalization map C(structure) . This operation implicitly defines a structure-dependent canonical permutation matrix **P**_can_ ∈ {0, 1}^*N*×*N*^ that rearranges the raw input into the canonical frame.

Under a raw reindexing **P**, the new canonical sorting matrix transforms as **P**_can_^′^ = **P**_can_ **P** ^T^. By Lemma 1, the resulting multichannel canonical interaction grid **X**_grid_^can^ is strictly invariant to the raw listing order up to machine precision:

10 $$X_{\mathrm{grid}}^{\mathrm{can}\prime }=M_{\mathrm{grid}}\left(C\left(P\cdot \mathrm{structure}\right)\right)=X_{\mathrm{grid}}^{\mathrm{can}}$$

The feature extraction network $M_{\mathrm{CNN}}$ consists of sequential 3 × 3 convolutional layers, batch normalization (BN), and activation functions σ. A 3 × 3 convolution applies a bank of spatial kernels $W_{k}\in R^{C_{\mathrm{out}}\times C_{\mathrm{in}}\times 3\times 3}$ over the structured grid. Because $M_{\mathrm{CNN}}$ acts directly on the canonically invariant grid, the extracted hidden feature grid **H**_grid_^can^ = $M_{\mathrm{CNN}}$ ­(**M**_grid_^can^) ∈ $R^{B\times C_{\mathrm{feat}}\times N\times N}$ inherits strict structural invariance:

11 $$H_{\mathrm{grid}}^{\mathrm{can}\prime }=M_{\mathrm{CNN}}\left(X_{\mathrm{grid}}^{\mathrm{can}\prime }\right)=M_{\mathrm{CNN}}\left(X_{\mathrm{grid}}^{\mathrm{can}}\right)=H_{\mathrm{grid}}^{\mathrm{can}}$$

Localized scalar canonical node features $H_{\mathrm{node}}^{\mathrm{can}}\in R^{N\times C_{\mathrm{feat}}}$ are obtained by extracting the diagonal elements (*i* = *j*) of the invariant feature grid. To feed these attributes into the downstream graph neural network alongside the original coordinates, they must be unsorted back into the raw input indexing sequence using the inverse canonical permutation **P**_can_^T^:

12 $$H_{\mathrm{node}}=P_{\mathrm{can}}^{T}H_{\mathrm{node}}^{\mathrm{can}}$$

Under a raw permutation **P**, the unsorted node feature matrix transforms as

13 $$H_{\mathrm{node}}^{\prime }={\left(P_{\mathrm{can}}^{\prime }\right)}^{T}H_{\mathrm{node}}^{\mathrm{can}\prime }={\left(P_{\mathrm{can}}P^{T}\right)}^{T}H_{\mathrm{node}}^{\mathrm{can}}={\mathrm{PP}}_{\mathrm{can}}^{T}H_{\mathrm{node}}^{\mathrm{can}}={\mathrm{PH}}_{\mathrm{node}}$$

yielding exact node-level permutation equivariance for the CNN feature extraction pipeline.

Let $G\left(H_{\mathrm{node}},R\right)$ represent the downstream message-passing graph neural network predicting atomic scalar magnetic moments $\mu \in R^{N\times 1}$ , where $R\in R^{N\times 3}$ is the raw 3D atomic coordinate matrix. Since G is constructed from standard node-permutation equivariant layers, it follows that

14 $$\mu^{\prime }=G\left({\mathrm{PH}}_{\mathrm{node}},\mathrm{PR}\right)=PG\left(H_{\mathrm{node}},R\right)=P\mu$$

Thus, despite the spatial neighbor-mixing behavior of the 3 × 3 convolutions, preprocessing the input with the canonical ordering frame followed by an inverse index projection ensures that an arbitrary raw permutation **P** results in the identical permutation applied to the output atomic magnetic moments **μ**.

#### 2.2.3 Numerical Proof

We applied a random permutation matrix **P** to re-index all constituent atoms of a six-atom (AmO_2_)_2_, then applied C to recover a canonical ordering before feeding the structure through the full pipeline, including the 3 × 3 convolutional branch. We compared predicted total energies (*E*, *E*_perm_) and analytical forces (**F** = −∇_**R**_ E, **F**_perm_ = −∇_**R**_perm__ *E*_perm_) between the original structure and the permuted structure. As shown below, energy invariance and force equivariance hold to single-precision floating-point limits (float32 machine precision) under the canonical-ordering hypothesis at this generic geometry:

15 $${\mathrm{Error}}_{E}=\left|E\left(R\right)-E\left(C\left(\mathrm{PR}\right)\right)\right|\approx 2.23\times 10^{-9}\;\mathrm{eV}$$

16 $${\mathrm{Error}}_{F}=\max_{i}∥F{\left(R\right)}_{i}-{\left(P^{T}F\left(C\left(\mathrm{PR}\right)\right)\right)}_{i}∥\approx 9.57\times 10^{-7}\;\mathrm{eV/Å}$$

### 2.3 Radial Basis Expansion

Interatomic periodic distances (*r*_*ij*_ = ∥**D**_*ij*_∥) within a fixed spherical boundary cutoff radius (*d*_max_ = 5.0 Å) are expanded into continuous, multichannel representations to establish smooth structural tracking without edge discontinuities. The radial basis function (RBF) mapping transforms these spatial distances using *N*_rbf_ = 32 centered Gaussian kernels

17 $${\mathrm{RBF}}_{k}\left(r_{\mathrm{ij}}\right)=\exp\left(-\gamma {\left(r_{\mathrm{ij}}-\mu_{k}\right)}^{2}\right)$$

where the kernel centers μ_*k*_ are uniformly distributed across the interval [0.0, *d*_max_] and the spatial scaling resolution parameter γ is explicitly defined as

18 $$\gamma ={\left[{\left(\frac{d_{\max}}{N_{\mathrm{rbf}}}\right)}^{2}\right]}^{-1}$$

This multichannel encoding is passed directly down to the radial network to generate the mixing weight coefficients for the geometric edge convolutions.

### 2.4 *E*(3)-Equivariant Geometric Graph Block

#### 2.4.1 Initial Structural Node States

The initial invariant structural node state for atom *i* is constructed by concatenating its learnable baseline chemical identity embedding with the atom-centered electronic spin descriptor extracted from the diagonal components of the 2D CNN matrix images:

19 $$h_{i}^{\left(0\right)}=\left[E_{\mathrm{chem}}\left(Z_{i}\right)∥h_{i}^{\mathrm{spin}}\right]\in R^{80\times 0e}$$

where $E_{\mathrm{chem}}\left(Z_{i}\right)\in R^{64}$ maps the atomic number *Z*_*i*_ to a chemical feature space, $h_{i}^{\mathrm{spin}}\in R^{16}$ represents the local spin configuration extracted via CNN­(**X**)_*m*,:,*a*,*a*_, and ∥ denotes channelwise concatenation.

#### 2.4.2 Periodic Geometric Edge Messages

To account for periodic boundary conditions, the true minimum-image displacement vectors **r**_*ij*_ = **D**_*ij*_ are mapped onto the irreducible representations (irreps) of the *O*(3) geometric point group under inversion parity using a basis of spherical harmonics up to angular momentum *l*_max_ = 2

20 $$Y\left({\mathrm{r̂}}_{\mathrm{ij}}\right)\in 1\times 0e⊕1\times 1o⊕1\times 2e$$

Directional edge messages **m**_*ij*_ are compiled via a fully connected tensor product. This operation utilizes the entire 80-dimensional initial node feature tensor **h**_*i*_^(0)^ from the source atom and modulates the angular interactions using radial weights **W**_*ij*_ derived from projecting the periodic distance ∥**r**_*ij*_∥ through an RBF network and a multilayer perceptron (MLP_radial_):

21 $$m_{\mathrm{ij}}=Y\left({\mathrm{r̂}}_{\mathrm{ij}}\right){⊗}_{W_{\mathrm{ij}}\left(\mathrm{RBF}\right)}h_{i}^{\left(0\right)}\in 32\times 0e⊕16\times 1o⊕8\times 2e$$

#### 2.4.3 High-Order Many-Body Correlations

Local atomic environments are accumulated by a topological pooling summation over the neighbor list N(i) , producing the pooled single-message tensor $A_{i}=\sum_{j\in N\left(i\right)}m_{\mathrm{ij}}$ . Higher-order many-body interactions are captured via a symmetric self-interaction tensor product parameterized by shared weights **W**_shared_:

22 $$B_{i}=A_{i}{⊗}_{W_{\mathrm{shared}}}A_{i}\in 64\times 0e⊕32\times 1o⊕16\times 2e$$

#### 2.4.4 Equivariant Mixing and Decoupled Readout

The many-body correlation tensor **B**_*i*_ is directly concatenated with the initial scalar invariants **h**_*i*_^(0)^ to assemble the full structural representation space **X**_*i*_ ∈ 144 × 0*e* ⊕ 32 × 1*o* ⊕ 16 × 2*e*. An equivariant linear layer projects this representation down to a fixed scalar and vector mixing space:

23 $${\mathrm{X̃}}_{i}=L_{\mathrm{equivariant}}\left(X_{i}\right)\in 128\times 0e⊕32\times 1o$$

Finally, the true scalar invariant block (**X̃**_*i*_ |_0*e*_ ≡ **X̃**_*i*_[:, : 128]) is cleanly isolated from directional vector components and mapped through decoupled MLP property heads to predict atomic energy contributions ε_*i*_ and local magnetic moments μ_*i*_:

24 $$\varepsilon_{i}={\mathrm{MLP}}_{\mathrm{energy}}\left({\mathrm{X̃}}_{i}{|}_{0e}\right),,\mu_{i}={\mathrm{MLP}}_{\mathrm{magmom}}\left({\mathrm{X̃}}_{i}{|}_{0e}\right)$$

The total potential energy of the periodic system is computed via a global pooling sum over all atoms within the cell context, *E*_total_ = ∑_*i*_ ε_*i*_.

### 2.5 Decoupled Multi-Task Property Readout and Analytical Force Derivation

The invariant structural features and high-order many-body correlation blocks are combined and linearly mixed via an equivariant projection to form the final multitask descriptor space:

25 $$h_{i}^{\mathrm{mixed}}=L_{\mathrm{equivariant}}\left([h_{i}^{\left(0\right)}∥B_{i}]\right)\in 128\times 0e⊕32\times 1o$$

The pure 0*e* scalar channels, spanning the first 128 dimensions of the representation, are cleanly isolated from the directional vector fields and mapped to independent, decoupled MLP readout heads to evaluate local atomic conditions:

26 $$\varepsilon_{i}={\mathrm{MLP}}_{\mathrm{energy}}\left(h_{i}^{\mathrm{mixed}}{|}_{0e}\right),\mu_{i}={\mathrm{MLP}}_{\mathrm{magmom}}\left(h_{i}^{\mathrm{mixed}}{|}_{0e}\right)$$

where **h**_*i*_^mixed^|_*0e*_ ≡ **h**_*i*_^mixed^[:, : 128].

The absolute potential energy (*E*_total_) of a periodic cell containing *N* atoms is reconstructed by accumulating local bonding energy contributions across global graph pooling operations and re-introducing the precomputed isolated atomic reference baselines (*ε*_base_):

27 $$E_{\mathrm{total}}=\sum_{i=1}^{N}\varepsilon_{i}+\sum_{i=1}^{N}\varepsilon_{\mathrm{base}}\left(Z_{i}\right)$$

where ε_base_(*Z*_*i*_) represents the static baseline shift for element *Z*_*i*_ calculated via ridge regression during dataset initialization.

Finally, conservative atomic force vectors **F**_*i*_ are exactly evaluated by computing the negative analytical gradient of the total energy expression with respect to the continuous nuclear Cartesian coordinates **R**_*i*_ using reverse-mode automatic differentiation:

28 $$F_{i}=-\nabla_{R_{i}}E_{\mathrm{total}}=-\frac{\partial E_{\mathrm{total}}}{\partial R_{i}}$$

To preserve the exact physical connection across periodic boundaries, the differentiation graph maps through the relative minimum-image displacements (**D**_*ij*_) explicitly during backpropagation:

29 $$D_{\mathrm{ij}}=\left(R_{j}-R_{i}\right)+S_{\mathrm{ij}}H$$

where **S**_*ij*_ is the periodic image translation vector and **H** is the cell lattice matrix, ensuring strict conservation of energy and momentum during structural evaluation.

#### 2.5.1 Dual-Pathway Analytical Gradient and Force Conservation

To rigorously evaluate energy conservation and prove that predicted forces are path independent, we derive the exact spatial gradient of *E*_total_ with respect to atomic position **R**_*k*_. The isolated atomic baselines *ε*_base_(*Z*_*i*_) are static species constants whose spatial gradients vanish identically (∇_**R**_*k*__ ε_base_ = **0**). Applying the multivariate chain rule to *E*_total_ = ∑_*i*_ ε_*i*_ yields the initial force expression:

30 $$F_{k}=-\nabla_{R_{k}}E_{\mathrm{total}}=-\sum_{i=1}^{N}\left(\frac{\partial \varepsilon_{i}}{\partial h_{i}^{\mathrm{mixed}}{|}_{0e}}\right)\cdot \left(\frac{\partial h_{i}^{\mathrm{mixed}}{|}_{0e}}{\partial R_{k}}\right)$$

Atomic coordinates **R**_*k*_ determine the total energy through two distinct, concurrent spatial pathways: the pairwise interatomic displacement vectors **D**_*ij*_ = (**R**_*j*_ – **R**_*i*_) + **S**_*ij*_ **H** within the graph topology, and the spatial screening factors exp­(−∥**D**_*ij*_∥) feeding the interaction grid channels of $M_{\mathrm{CNN}}$ . Differentiating **D**_*ij*_ with respect to **R**_*k*_ yields

31 $$\frac{\partial D_{\mathrm{ij}}}{\partial R_{k}}=\delta_{\mathrm{jk}}I_{3}-\delta_{\mathrm{ik}}I_{3}$$

where δ_*ab*_ is the Kronecker delta and **I**_3_ is the 3 × 3 identity matrix. Note that while **R**_*k*_ also determines the canonical ordering permutation matrix **P**_can_ via the secondary key *d*_*i*_, this sorting operation is a piecewise-constant discrete mapping whose derivative vanishes almost everywhere (∂**P**_can_/∂**R**_*k*_ = **0**). PyTorch autograd thus routes the exact analytical force on atom *k* strictly across both continuous spatial tracks:

32 $${\mathrm{F⃗}}_{k}=-\sum_{i,j}\left(\frac{\partial E_{\mathrm{total}}}{\partial h_{\mathrm{graph}}}\frac{\partial h_{\mathrm{graph}}}{\partial D_{\mathrm{ij}}}+\frac{\partial E_{\mathrm{total}}}{\partial X_{\mathrm{grid}}^{\mathrm{can}}}\frac{\partial X_{\mathrm{grid}}^{\mathrm{can}}}{\partial ∥D_{\mathrm{ij}}∥}\frac{\partial ∥D_{\mathrm{ij}}∥}{\partial D_{\mathrm{ij}}}\right)\cdot \left(\delta_{jk}-\delta_{ik}\right)I_{3}$$

Regrouping terms over pairs (*k*, *j*) and (*i*, *k*) by executing the Kronecker summations, the pairwise expressions collapse cleanly into a conservative, symmetric closed-form analytical force vector:

33 $$F_{k}=\sum_{j\in N\left(k\right)}\left(\frac{\partial \varepsilon_{k}}{\partial h_{k}^{\mathrm{mixed}}{|}_{0e}}\frac{\partial h_{k}^{\mathrm{mixed}}{|}_{0e}}{\partial D_{\mathrm{kj}}}-\frac{\partial \varepsilon_{j}}{\partial h_{j}^{\mathrm{mixed}}{|}_{0e}}\frac{\partial h_{j}^{\mathrm{mixed}}{|}_{0e}}{\partial D_{\mathrm{jk}}}\right)$$

Strict energy conservation and physical consistency follow directly from two key structural properties of this differentiation graph:

1. **Path independence and zero curl:** Because **F**_*k*_ is evaluated purely as the negative analytical gradient of a single-valued, continuous scalar energy functional *E*_total_(**R**), the curl of the force field vanishes identically everywhere (∇ × **F** = **0**). Consequently, any closed trajectory line integral satisfies ∮ **F**·d**R** = 0, guaranteeing exact analytical conservativeness of the force field during microcanonical (NVE) molecular dynamics simulations.
2. **Absence of implicit magnetic derivative coupling:** At any given simulation step *t*, input magnetic moments **μ** ^(*t*–1)^ act as fixed physical state descriptors inherited from the previous step. Updated magnetic moments **μ̂**^(*t*)^ for step *t* + 1 are predicted simultaneously via an independent, decoupled multitask readout head MLP_magmom_. Because input moments **μ** ^(*t*–1)^ are treated as fixed non-differentiable features during force computation, implicit derivative coupling terms vanish identically:
34 $$\sum_{i=1}^{N}\frac{\partial E_{\mathrm{total}}}{\partial \mu_{i}^{\left(t-1\right)}}\frac{\partial \mu_{i}^{\left(t-1\right)}}{\partial R_{k}}\equiv 0$$
  This explicit structural decoupling ensures that the evolution of magnetic degrees of freedom introduces no artificial torque, energy drifts, or non-conservative phantom forces into the molecular dynamics engine.

## 3 Data Pipeline and Feature Engineering

A fundamental challenge in designing multimodal machine learning interatomic potentials is the synchronized scaling of dense, grid-based topological descriptors alongside sparse, message-passing structural graphs. This coupling must be achieved without introducing memory leaks or corrupting the computational graph required for analytical backpropagation. We resolve this structural bottleneck by engineering a customized data collation pipeline that maps absolute electronic configurations onto graph nodes while dynamically compiling the neighborhood topology.

### 3.1 Static Topology Caching and Periodic Neighborhood Alignment

To eliminate the massive computational overhead of evaluating periodic neighbor lists during training, we implement an asynchronous topology caching pipeline. The neighborhood structure is resolved exactly once per system configuration during dataset initialization, decoupling the heavy geometric sorting from the active optimization loops.

For periodic crystal systems, the boundaries are defined by a cell lattice matrix $H=\left[a,b,c\right]\in R^{3\times 3}$ . To capture exact interatomic displacements across these periodic cells, the displacement vectors **D**_*ij*_ are computed under the minimum image convention (MIC) via an optimized cell-linked neighbor list search:

35 $$D_{\mathrm{ij}}=R_{j}-R_{i}-H\cdot \left\lfloor H^{-1}\left(R_{j}-R_{i}\right)+\frac{1}{2}1\right\rfloor$$

where **R** represents absolute nuclear coordinates, and ⌊·⌋ denotes the elementwise floor operation that transforms absolute distances into fractional space to map the nearest periodic image. The active periodic graph edge topology E is bounded by a strict cutoff radius of *r*_cut_ = 5.0 Å:

36 $$E=\left\{\left(i,j\right)\;|\;∥D_{\mathrm{ij}}∥\leq r_{\mathrm{cut}},i\neq j\right\}$$

During the data collation stage, individual graph structures are fused into a rigid, disjoint global mini-batch container. To preserve the computational graph for analytical backpropagation, the continuous absolute position coordinates are explicitly tracked as leaf nodes (**R** ∈ graph.pos).

To prevent cross-talk between separate systems in the mini-batch, the global edge index mapping shifts individual atom pointers using a batch allocation vector **b**, where **b**_*i*_ = **b**_*j*_ enforces strict intra-molecular connectivity. Finally, during the training forward pass, the dynamic coordinate gradients (∇_**R**_*i*__ *E*_total_) are re-anchored to the cached periodic displacements by combining the open-space variations with the static out-of-cell wrapping offsets:

37 $$r_{\mathrm{ij}}=\left(R_{\mathrm{dst}}-R_{\mathrm{src}}\right)+\left(D_{\mathrm{ij}}-\left(R_{\mathrm{dst},\mathrm{static}}-R_{\mathrm{src},\mathrm{static}}\right)\right)$$

This formulation preserves the exact derivative chain rule through the periodic boundaries on parallel graphics hardware without requiring slow, real-time graph re-compilation loops.

### 3.2 Multi-Task Optimization and Joint Loss Function

To simultaneously optimize the network for potential energy surfaces, conservative atomic force fields, and localized electronic magnetic moments, we implemented a joint multitask loss objective function. Absolute potential energies scale extensively with system size, thus, direct minimization introduces severe numerical bias toward larger molecular structures. We eliminate this scaling artifact by normalizing the shifted bonding energies to an intensive per-atom basis. The localized structural bonding energy loss component is formulated as

38 $$L_{\mathrm{energy}}=\frac{1}{B}\sum_{b=1}^{B}{\left(\frac{\Delta E_{\mathrm{pred},b}}{N_{b}}-\frac{\Delta E_{\mathrm{true},b}}{N_{b}}\right)}^{2}$$

where *B* denotes the mini-batch size, *N*_*b*_ represents the total atom count in configuration *b*, and Δ*E* corresponds to the isolated bonding energy contribution after removing the non-differentiable single-atom baseline shifts.

Conversely, atomic force vectors and magnetic moments operate as localized, atom-centered properties that do not scale with cell volume and do not require extensive normalization. To prevent zero-padded background dummy elements from introducing artificial gradients during batch calculations, a binary structural masking filter ( $M_{i}=I\left(Z_{i}>0\right)$ ) is applied across the total pool of batched atoms. The corresponding localized force vector and magnetic moment magnitude loss functions are defined using mean square error (MSE) metrics:

39 $$L_{\mathrm{force}}=\frac{1}{\sum_{i}M_{i}}\sum_{i}M_{i}{∥F_{i}-F_{i,\mathrm{true}}∥}^{2}$$

40 $$L_{\mathrm{magmom}}=\frac{1}{\sum_{i}M_{i}}\sum_{i}M_{i}{\left(\mu_{i}-\mu_{i,\mathrm{true}}\right)}^{2}$$

The network parameters are optimized simultaneously across the total multitask learning boundary using a weighted scalar objective framework:

41 $$L_{\mathrm{total}}=w_{E}L_{\mathrm{energy}}+w_{F}L_{\mathrm{force}}+w_{M}L_{\mathrm{magmom}}$$

The hyperparameter set utilized in this study are tabulated in supporting information (Table S1). This multitask weighting heavily prioritizes baseline energy surface convergence while retaining sufficient force field directional alignment to guarantee smooth, stable geometric optimization pathways during potential energy surface scans.

Finally, to prevent gradient explosions through highly expressive *E*(3) tensor product pathways during backpropagation, updating trajectories are regulated using an active norm constraint threshold applied to the global network parameters:

42 $${∥g∥}_{2}\leq 10.0$$

where **g** is the combined parameter gradient vector, stabilizing the deep linear mixing layers across highly disparate chemical environments.

## 4 Results and Discussion

We evaluate the predictive performance and physical transferability of KENJI through rigorous testing and validation on independent HE26 testing dataset which are as follows:

### 4.1 HE26 Composition, Data Splitting, and Overlap Analysis

To ensure strict reproducibility and direct comparability with MACE-osaka26 model, we adopt the official prepartitioned training and testing datasets provided in the public HE26 Figshare repository.^22^ HE26 dataset (Figure 2). The dataset encompasses a diverse spectrum of chemical complexity across heavy element systems, categorized into elemental solids, binary oxides, multicomponent (quaternary+: four or more species) fluorite oxides, and actinide organometallic molecular complexes.

**Figure 2:**
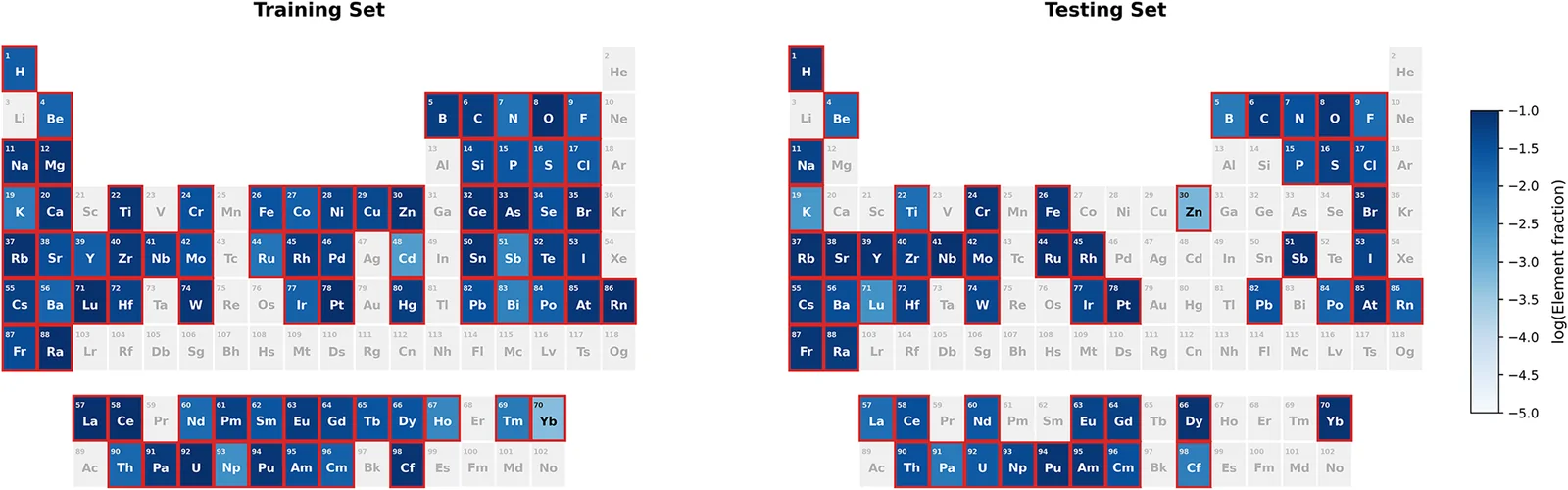
Atomic count breakdown of HE26 training and testing set.

As illustrated in Figure 3, both the training and evaluation subsets exhibit proportional distributions across chemical complexity domains, dominated by quaternary+ heavy-element configurations. Fluorite oxides, which dominate the dataset, comprise binary-to-quinary mixed oxides of actinides, lanthanides, and rare earth elements, targeting actinide-based high entropy ceramics and minor-actinide mixed oxide fuels.

**Figure 3:**
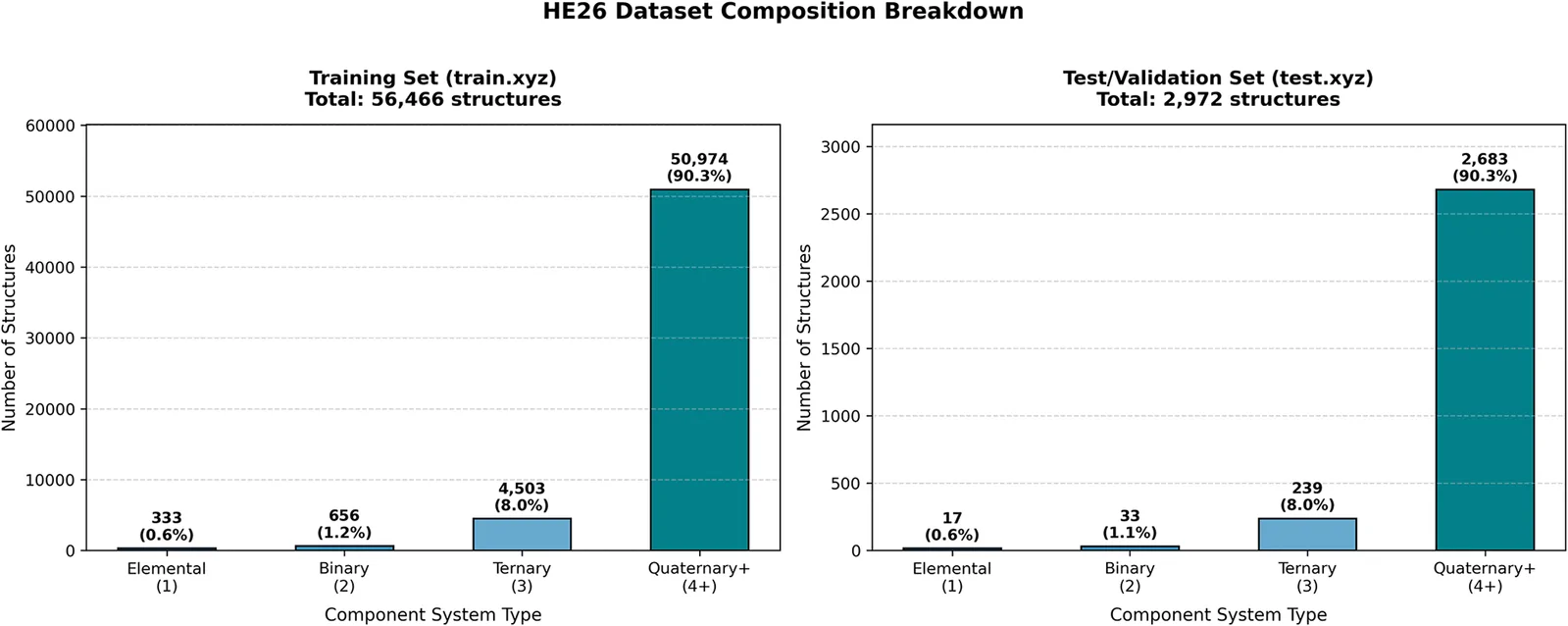
Compositional breakdown and system-type distribution across the official HE26 training and testing set, categorized by unique atomic species per structure.

#### 4.1.1 Structural Redundancy

To rigorously audit data split, we implemented a canonical spatial geometry fingerprinting check. Structures were hashed using MD5 strings encoding sorted atomic species, periodic cell vectors, and wrapped fractional positions evaluated at a spatial precision threshold of 10^–4^ Å. This spatial fingerprinting revealed 117 identical geometric configurations shared directly between the official training set and the testing set. However, to maintain rigorous standards while preserving benchmark comparability, and given the low percentage of redundancy, we evaluate our model performance under the *Official HE26 Split.*

### 4.2 Multi-Task Training Convergence and Generalization Performance

KENJI was optimized over a multitask learning boundary targeting bonding energies, conservative atomic force fields, and localized electronic magnetic moments simultaneously. Training was conducted for 100 epochs on the HE26 dataset under an automated learning rate scheduler (Table S1 for detail overview of hyperparameters), achieving stable and monotonic convergence without experiencing numerical drift, singular behavior, or gradient explosions.

Figure 4 shows the training parity plots for energy, forces, and magnetic moments. The network converged to a localized bonding energy mean absolute error (MAE) of 6.93 meV/atom (RMSE = 13.28 meV/atom), whereas the MACE-osaka26 model gives MAE of 44.7 meV/atom (RMSE = 117.3 meV/atom). The conservative atomic force MAE of 34.10 meV/Å (RMSE = 66.51 meV/Å) is on par with values from the MACE-osaka26 model (26.30 meV/Å, RMSE = 73.70 meV/Å). Concurrently, the atom-centered electronic magnetic moments converged to a tight error bound of 0.0257 μ_B_. This co-dependent convergence of energy, force, and magnetic-moment errors indicates that the binary structural masking filter ( $M_{i}=I\left(Z_{i}>0\right)$ ) effectively excludes zero-padded dummy atoms from contributing spurious gradients during training.

**Figure 4:**
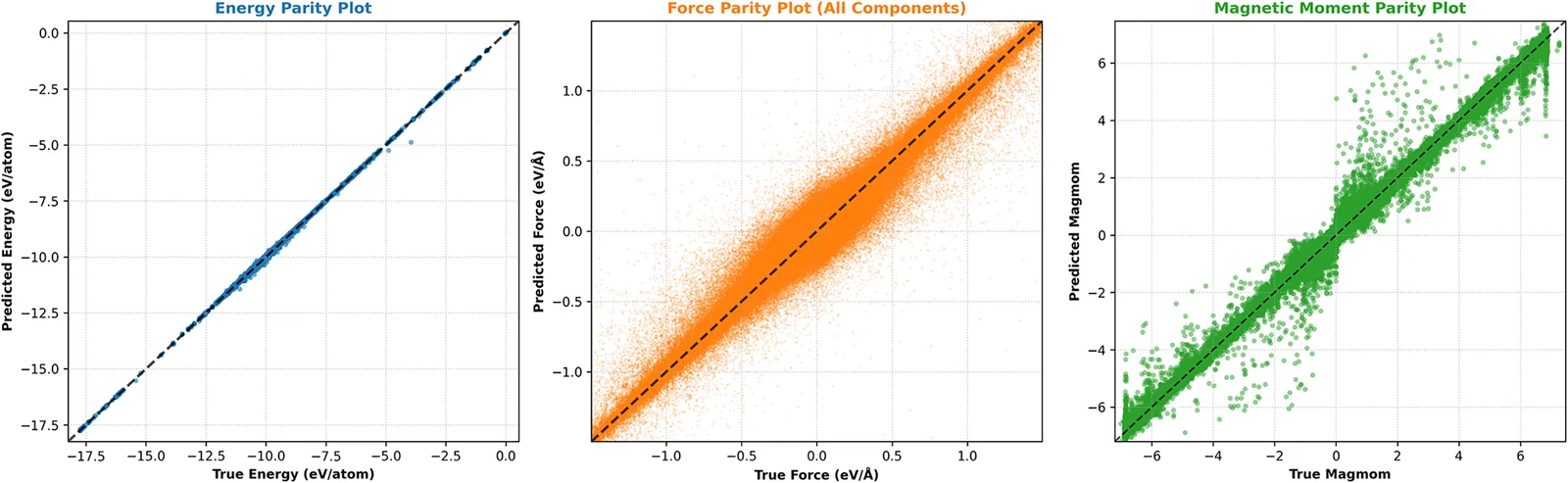
Comprehensive training metrics on the HE26 training dataset for intensive bonding energy (meV/atom), local atomic force vector field (meV/Å), and localized magnetic moments (μ_B_).

To rigorously evaluate the predictive transferability and out-of-sample generalization capacity of our model, it was deployed against an independent evaluation on HE26 testing systems. Absolute potential energies were reconstructed by re-introducing the static, unshifted elemental reference baselines derived from our initial ridge-regression matrix framework.

Table 1 and Figure 5 demonstrates that the testing metrics of MACE-osaka26 model (values in the bracket), especially for force predictions, outperforms our MLIPs. One factor is the inclusion of stress in the training. MACE based model is trained with *w*_*E*_ = 1.0, *w*_*F*_ = 10.0, and *w*_stress_ = 100, whereas current KENJI is not trained on stress at all. The close agreement between the models indicates the importance of stress based input features for MLIP generation, which will be included in the future versions of our model. Moreover, KEN establishes itself as a competitive architecture for generating MLIPs with multi modal input flexibility.

**Table 1:** KEN Validation Metrics Evaluated Over HE26 Independent Testing Configurations

descriptor	MAE^a^	RMSE^b^
*E*_total_ (meV/atom)	46.74 (48.00)	163.10 (123.51)
*F⃗* (meV/Å)	51.93 (27.82)	140.19 (72.06)
μ	0.0629 μ_B_	0.2141 μ_B_

a MAE: mean absolute error.

b RMSE: root-mean-square error.

**Figure 5:**
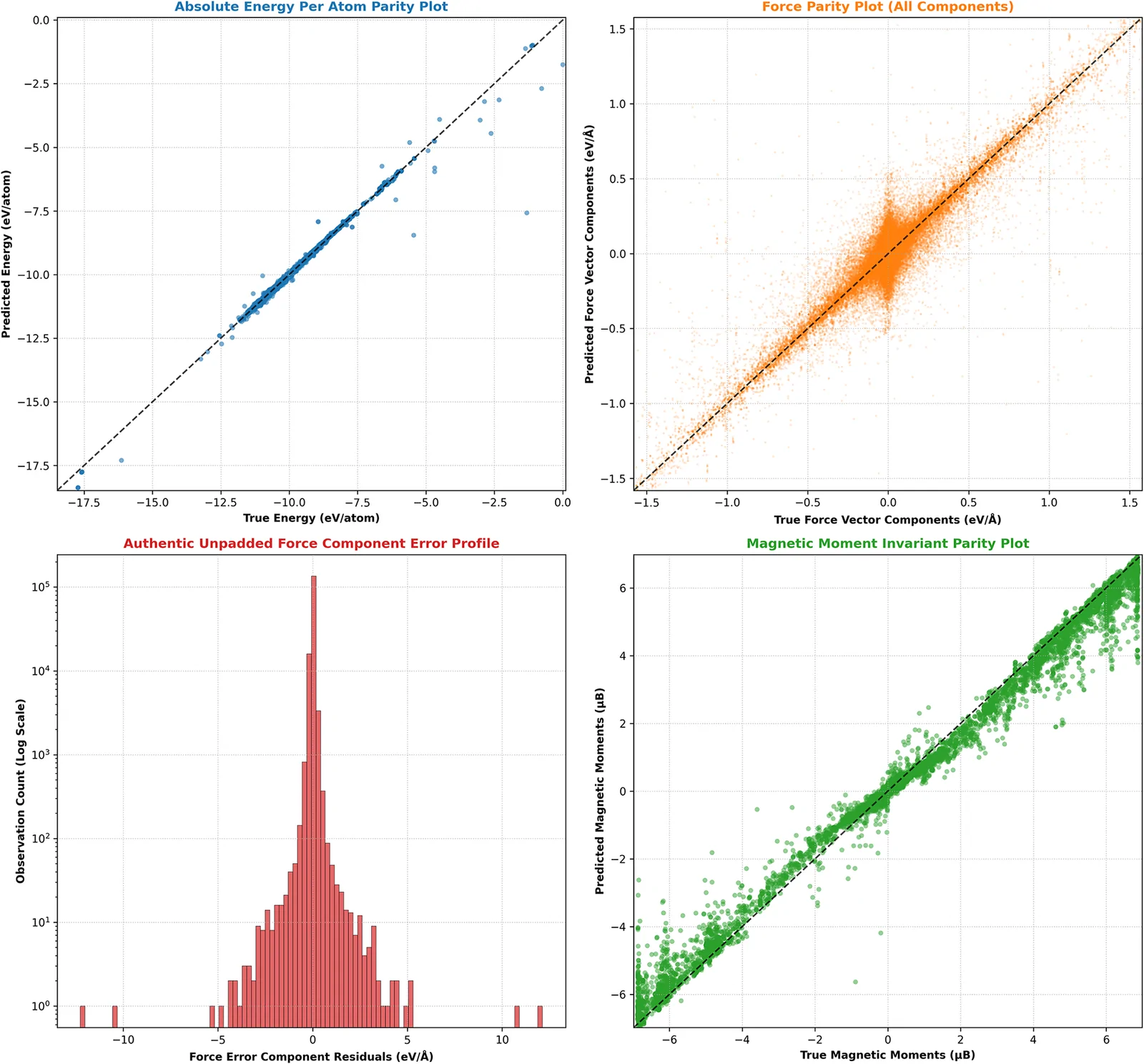
Comprehensive testing metrics on the HE26 testing dataset for intensive bonding energy (meV/atom), local atomic force vector field (meV/Å), and localized magnetic moments (μ_B_).

### 4.3 Benchmarking Against CHGNet magnetic MLIP

To evaluate the capacity of the KENJI to capture electronic spin configurations alongside spatial potential energy surfaces, we examine the predicted versus reference absolute magnetic moments (|μ|) over 648 structures randomly picked from the testing dataset as CHGNet is limited to predicting the magnitude (|μ|) only and up to Pu atom. We compare the baseline performance of CHGNet against the KENJI with 50% perturbed DFT reference values as the input guess. The quantitative prediction performance metrics are summarized in the parity subplots in Figure 6. Scatter distributions reveal contrasting physical learning mechanics between the two architectures.

**Figure 6:**
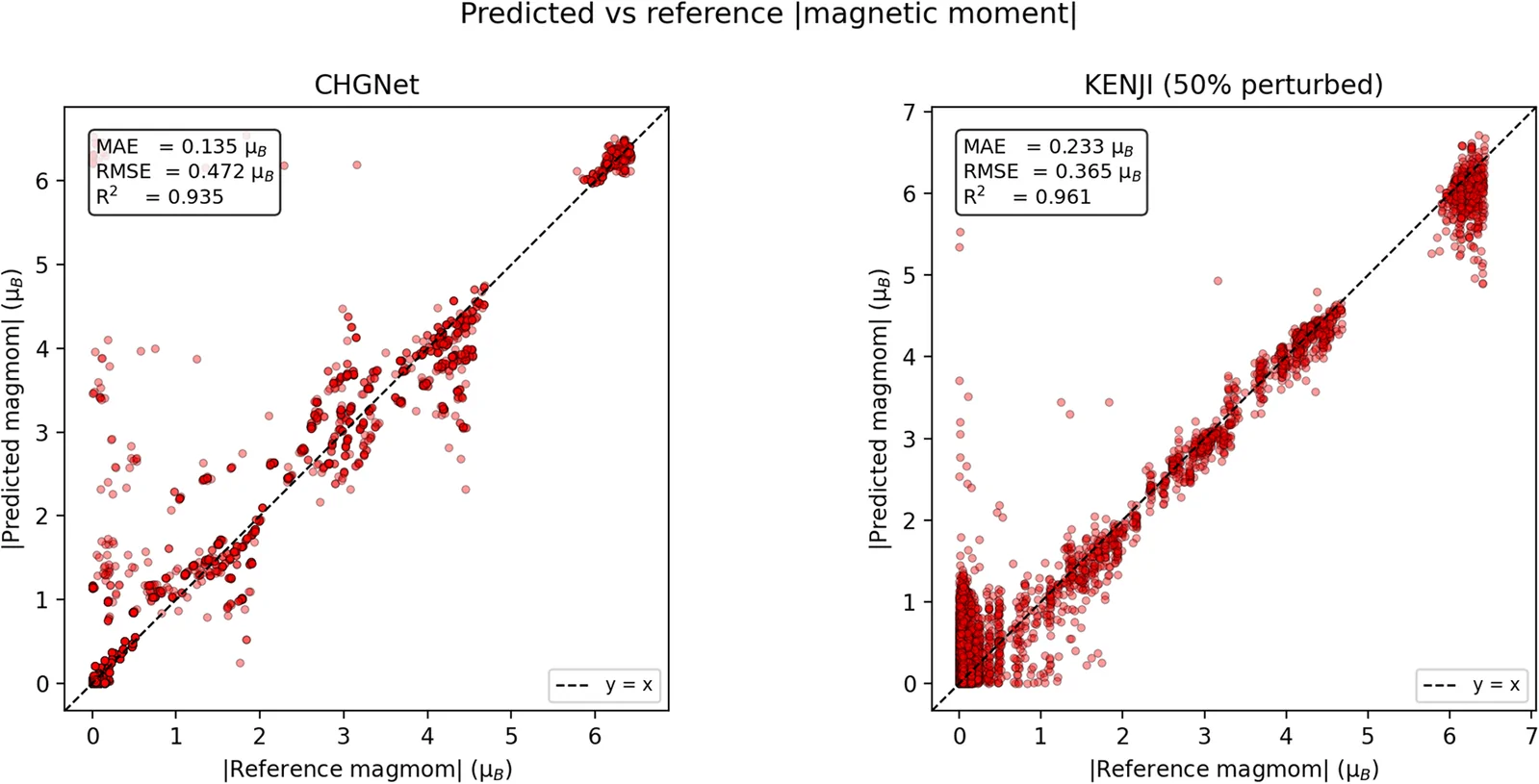
Parity plots of predicted versus reference absolute magnetic moments (|magmom|) for (left) CHGNet and (right) KENJI (50% perturbed). The dashed line represents the ideal *y* = *x* tracking baseline.

#### 4.3.1 CHGNet Localization Profiles

CHGNet demonstrates tighter local tracking in the 0.0 μ_B_ < |μ| < 2.5 μ_B_ spin range, with a low overall baseline MAE of 0.135 μ_B_. However, it suffers from noticeable under-prediction and higher variance scatter in 3.0 to 4.5 μ_B_ range, leading to a higher RMSE of 0.472 μ_B_.

#### 4.3.2 KENJI Geometric Alignment

Conversely, the KENJI (50% perturbed) configuration yields a highly linear alignment tightly adhering to the ideal *y* = *x* diagonal across the entire range up to 7.0 μ_B_. This structurally uniform tracking yields a significantly higher *R* ^2^ = 0.961 and reduces catastrophic outliers, producing a lower overall RMSE of 0.365 μ_B_. The high-density vertical accumulation of data points near 0.0 μ_B_ indicates KENJI introduces a localized background noise floor in non-magnetic or weakly symmetric local crystal environments. This localized noise contributes to the higher overall MAE 0.233 μ_B_ despite maintaining superior linear correlation across the rest of the chemical space.

### 4.4 Characterizing the Importance of the CNN Spin Descriptor

The magnetic-moment branch of KEN extracts an atom-centered descriptor $h_{i}^{\mathrm{spin}}\in R^{16}$ from the diagonal of a 2D convolutional feature map. To precisely characterize what physical information **h**_*i*_^spin^ encodes, we performed a direct diagnostic analysis of the deployed model. Within a representative structure, we observe a strong linear relationship (Pearson correlation *r* = 0.975) between ∥**h**_*i*_^spin^∥ and each atom’s local magnetic moment μ_*i*_, confirming that local spin magnitude is the dominant determinant of the descriptor.

To test whether the descriptor captures multibody spin ordering—critical for describing the complex potential energy surfaces of actinides and strongly correlated systems—we simulated a local magnetic phase transition. In KEN, the input grid channels map pairwise alignments through a tanh­(μ_*i*_ μ_*j*_) kernel, assigning positive signatures to ferromagnetic (FM) orderings and negative signatures to antiferromagnetic (AFM) orderings. Holding all atomic positions fixed, we toggled an adjacent canonical oxygen pair (Atoms 0 and 1) from an FM state (μ_0_ = μ_1_ = +2.0 μ_B_) to an AFM state (μ_0_ = +2.0 μ_B_, μ_1_ = −2. 0 μ_B_).

As summarized in Table 2, the CNN branch demonstrates high sensitivity to local magnetic phase boundaries, registering a substantial feature-space perturbation on the modified atomic pair. The propagation profile also exposes the exact boundary of the network’s receptive field: the phase-transition signal propagates across precisely three adjacent matrix indices before decaying to *exactly zero* at Atom 4. This strict spatial truncation confirms that the feature extraction pipeline operates through two 3 × 3 convolutional layers with unit padding.

**Table 2:** Response of **h**_*i*_^spin^ across Atoms following a Localized FM-to-AFM Phase Transition between Matrix Indices 0 and 1

matrix index (*i*)	vector shift ∥Δ**h**_*i*_^spin^∥_2_	physical context
0	2.191	perturbed atom A (oxygen, *Z* = 8)
1	6.136	perturbed atom B (oxygen, *Z* = 8)
2	1.290	first-order matrix neighbor
3	0.336	receptive field boundary element
≥4	0.000 (exact)	unaffected matrix domain

Because the canonical map groups atoms primarily by elemental species and secondarily by radial distance, these 2D grid convolutions do not evaluate conventional 3D spatial neighbor shells. Instead, they act as highly structured filters capturing localized magnetic couplings within chemically homogeneous sub-lattices, which is crucial for magnetic actinide materials. This element-segregated indexing provides a regularized spin topology descriptor that standard geometric graph neural networks cannot natively construct in their early message-passing layers.

### 4.5 Computational Scalability and Supercell Transferability

#### 4.5.1 Theoretical and Empirical Scaling Complexity

KEN combines two structurally distinct components with different scaling behavior, and we clarify this distinction explicitly rather than characterizing the architecture with a single complexity class. The *E*(3)-equivariant geometric message-passing branch operates strictly on a local neighbor graph defined within a radial cutoff (*r*_cut_) and scales as O(N) , consistent with standard local graph-based interatomic potentials. The magnetic spin-descriptor branch, however, constructs a dense *N*_max_ × *N*_max_ pairwise interaction grid (matrix_images) processed by a 2D convolutional feature extractor; this component scales as $O\left(N^{2}\right)$ in both memory and compute, independent of convolutional kernel size, since the grid itself—not the convolution operation—is the source of the quadratic scaling. Thus, the two branches genuinely differ in their asymptotic behavior.

##### 4.5.1.1 Empirical Timing Benchmark

We benchmarked wall-clock inference time (forces and energy) for fluorite AmO_2_ supercells ranging from *N* = 96 to 2592 atoms on ARM CPU. Over this range we observe a local scaling exponent (fit to time ∝ *N* ^*p*^ between consecutive benchmarked sizes) that is consistent with the increasing relative cost of the $O\left(N^{2}\right)$ spin-descriptor branch as *N* grows, as shown in Figure 7 (also Table S2).

**Figure 7:**
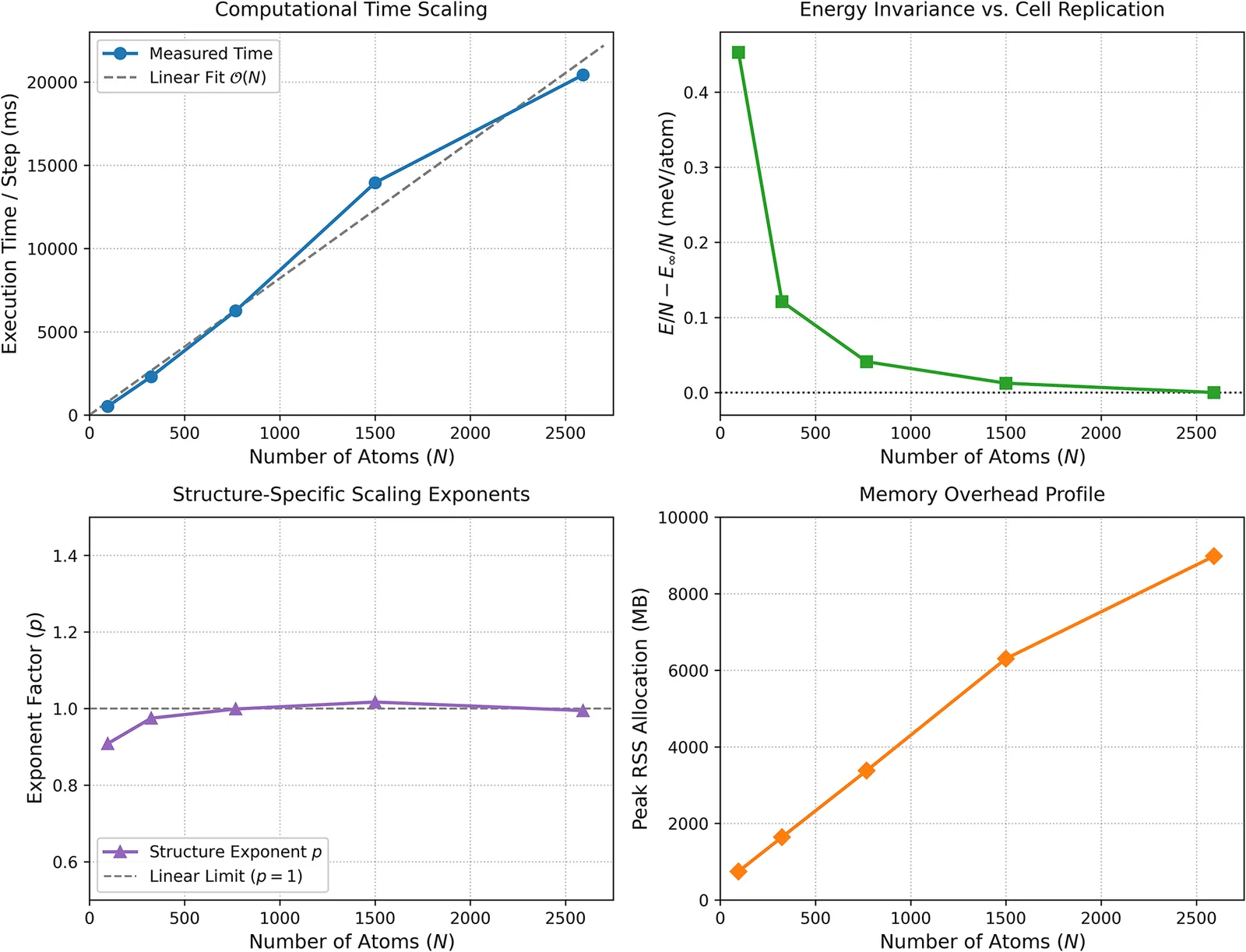
Computational scaling and cell replication invariance of the KENJI potential with single-step inference execution time as a function of atom count, and local scaling exponent p along with the CPU Memory scaling with increasing *N*.

#### 4.5.2 Energy Invariance

The interatomic potential is formulated as a sum of local atomic site energies *e*_*i*_. Thus, models trained on smaller atomic configurations are transferable to large supercells. Under periodic boundary conditions, cell replication preserves local atomic environments within *r*_cut_ across sub-lattices. As presented in Figure 7, the energy per atom demonstrates strict invariance upon cell replication, with a maximum variation bounded by Δ*E*_per atom_ ≤ 4.53 ×10^–4^ eV/atom across all supercell dimensions.

### 4.6 Equation-of-State Volume and Lattice Parameter Benchmark

To evaluate the accuracy of the learned potential energy surface near equilibrium geometry, we fitted the third-order Birch–Murnaghan^23^ equation of state (EOS) to KENJI-predicted energy–volume curves obtained under isotropic volume strains of ±5%, sampled at 9 points per structure, across the HE26 testing dataset. The MAE_*V*_0__ and RMSE_*V*_0__ values deviate by 47.6241 and 107.2874 Å^3^, respectively from the DFT+*U* reference data, whereas the corresponding equilibrium lattice parameter, derived from the fitted volume via the shape-agnostic isotropic-scaling relation *a*_0_ = *a*_original_(*V*_0_/*V*_original_)^1/3^, gives MAE_*a*_0__ and RMSE_*a*_0__ as 0. 3733 and 0. 5165 Å, respectively.

#### 4.6.1 Origin of the Largest Residuals

Inspection of the largest individual residuals identifies a consistent physical origin: the worst-fitting configurations correspond to molecular actinide-extractant coordination complexes (e.g., Am-DEHPA-type structures), which differ fundamentally from the extended ionic/covalent lattices. In these compounds, bulk compressibility under isotropic strain is governed largely by soft intermolecular packing modes rather than the stiff, isotropic lattice response the Birch–Murnaghan form assumes. This results in disproportionately large *V*_0_ residuals for this small subset of configurations relative to the ionic/covalent actinide crystals that dominate the benchmark set.

Upon separating the largest residuals, and re-evaluating, the MAE_*V*_0__^excl.^ and RMSE_*V*_0__^excl.^ goes down to 42.7764 and 71.8944 Å^3^, respectively with an improved *R*_excl._^2^ = 0.9425, confirming that the extended ionic/covalent actinide crystals are fitted substantially more accurately, and that the residual gap is attributable to a well-characterized, physically distinct structural class rather than to model inaccuracy. Figure 8 shows the corresponding parity plots for both quantities against the reference DFT+*U* values, with the molecular-crystal configurations discussed above visible as the small population of points furthest from the ideal 1:1 line.

**Figure 8:**
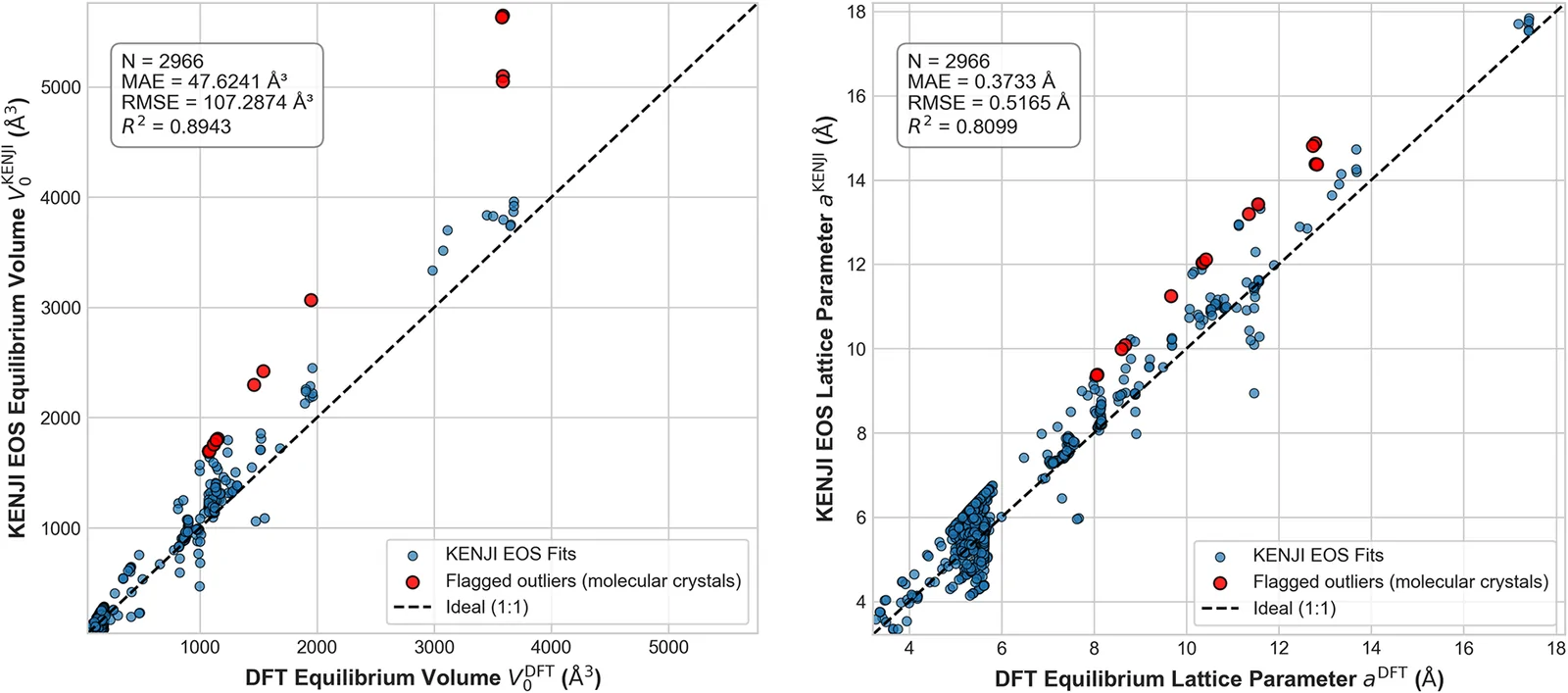
Parity between KENJI-fitted and reference DFT+*U* equilibrium volume *V*_0_ (left) and lattice parameter *a*_0_ (right) across all benchmarked HE26 validation configurations. The small population of points showing the largest deviation from the ideal 1:1 line correspond to molecular actinide-extractant coordination complexes (e.g., Am-DEHPA).

### 4.7 Microcanonical Energy Conservation and Gradient Consistency

To verify that the automatic differentiation graph (**F**_*i*_ = −∇_**r**_*i*__ *E*_total_) is strictly conservative, we performed NVE simulations at 300 K to allow enough thermal energy to explore the anharmonic areas of the PES. All NVE trajectories were evaluated over a production period of *t*_prod_ = 20.0 ps following an initial 2.0 ps (NVT) pre-equilibration phase using Langevin dynamics at 300 K. We quantify numerical performance using two primary metrics, namely, the linear energy drift rate (γ_*E*_, in units of meV/atom/ps) and the energy fluctuation ratio (η_*E*_):

43 $$\gamma_{E}=\frac{d}{dt}\left(\frac{E_{\mathrm{tot}}}{N}\right),,\eta_{E}=\frac{\sigma \left(E_{\mathrm{tot}}\right)}{\sigma \left(E_{\mathrm{kin}}\right)}$$

An η_*E*_ value of ≪1 signifies that total energy fluctuations are negligible compared to kinetic energy exchange, demonstrating proper microcanonical sampling

#### 4.7.1 Microcanonical (NVE) Stability and Data-Driven Refinement Validation

To ensure statistical rigor, and eliminate initialization bias, simulations were performed across three independent initial velocity distributions drawn from the Maxwell–Boltzmann distribution using different random seeds (seeds 42, 101, and 202) across three integration timesteps (Δ*t* ∈ {0.25, 0.5, 1.0} fs). We conducted benchmarks on non-periodic (UO_2_)_4_ cluster, as well as periodic CmO_2_. As summarized in Table 3 and Figure 9, the (UO_2_)_4_ cluster demonstrated near-perfect energy conservation across all timesteps and random initialization seeds, yielding absolute energy drift rates consistently well below 0.1 meV/atom/ps. However, for the periodic CmO_2_, we observed a localized conservation degradation, with energy drift peaking at 6.186 meV/atom/ps during the Δ*t* = 0.5 fs trials.

**Table 3:** Comprehensive Microcanonical (NVE) Conservation Tracking Metrics Comparing the Original KENJI Framework Responses on (UO_2_)_4_ Cluster and Periodic CmO_2_ Against the 1565-Structure Refined KENJI ^a^

		[UO_2_]_4_ (KENJI)		CmO_2_ (KENJI)		CmO_2_ (refined KENJI)	
step Δ*t* (fs)	seed	drift	fluct.	drift	fluct.	drift	fluct.
	42	–0.0063	0.215	1.6576	0.280	0.0016	0.058
0.25	101	–0.0255	0.041	1.4577	0.089	0.0571	0.302
	202	0.0146	0.163	–1.3794	0.212	0.0426	0.083
	42	–0.0316	0.350	6.1864	0.306	0.0661	0.190
0.50	101	0.0800	0.208	2.5208	0.264	0.1090	0.197
	202	–0.0108	0.065	–0.9671	0.255	0.1765	0.255
	42	–0.0413	0.052	0.0809	0.229	0.0809	0.229
1.00	101	0.0268	0.166	–0.0169	0.106	–0.0169	0.106
	202	–0.0407	0.058	4.6655	0.305	0.0050	0.076

a Drifts are reported in units of meV/atom/ps, alongside corresponding localized temperature fluctuation ratios.

**Figure 9:**
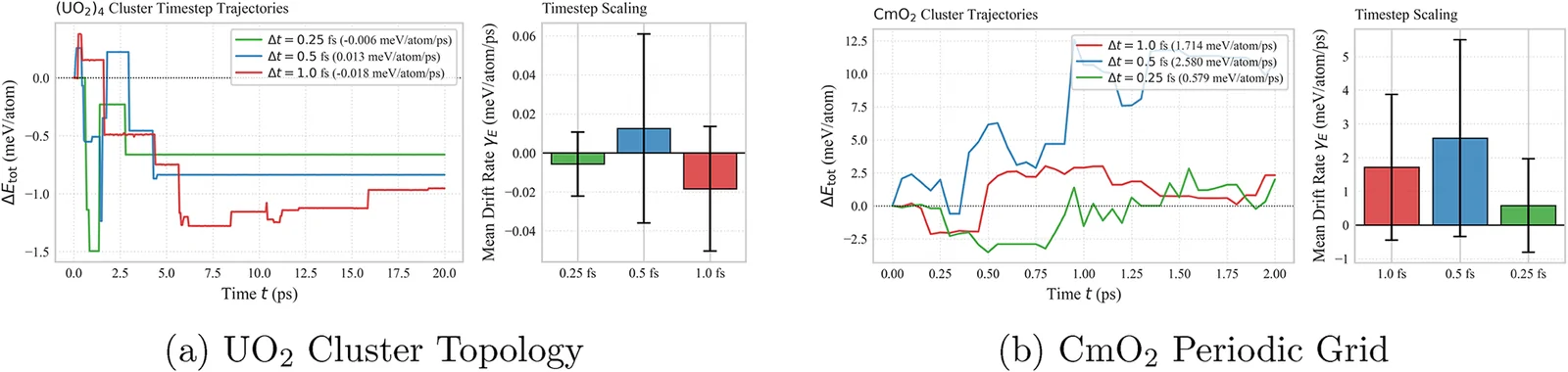
Total energy conservation tracking using KENJI model during (NVE) dynamics. (a) Near-perfect physical energy preservation for the (UO_2_)_4_ cluster across all timesteps (<0.05 meV/atom/ps). (b) Localized numerical degradation observed for the periodic CmO_2_, with drift spike peaking at 6.186 meV/atom/ps.

To determine whether this localized drift anomaly stemmed from an intrinsic limitation in the KEN architecture or an under-sampled regional potential energy surface, we refined our network weights by training exclusively on a 1565-structure subset (termed as AcHE26) of HE26 dataset. This specialized corpus imposes strict operational atom-count boundaries (*N* ≤ 50) and explicitly isolates actinide-specific correlation criteria spanning active element spaces (U, Np, Pu, Am, Cm, O, H, C, Eu, Zr, N, F, Cl).

Following this target refinement, the refined KENJI was re-subjected to the identical CmO_2_ simulation pathways. As reported in the third column block of Table 3, the refined model restored exceptional integration behavior across all timesteps, shifting the maximum absolute tracking drift downward to <0.18 meV/atom/ps while exhibiting ideal sub-femtosecond discretization scaling (Figure 10). This stark contrast definitively establishes that the initial simulation anomalies were entirely a function of training data coverage rather than architectural vulnerabilities within the KEN descriptor pipelines.

**Figure 10:**
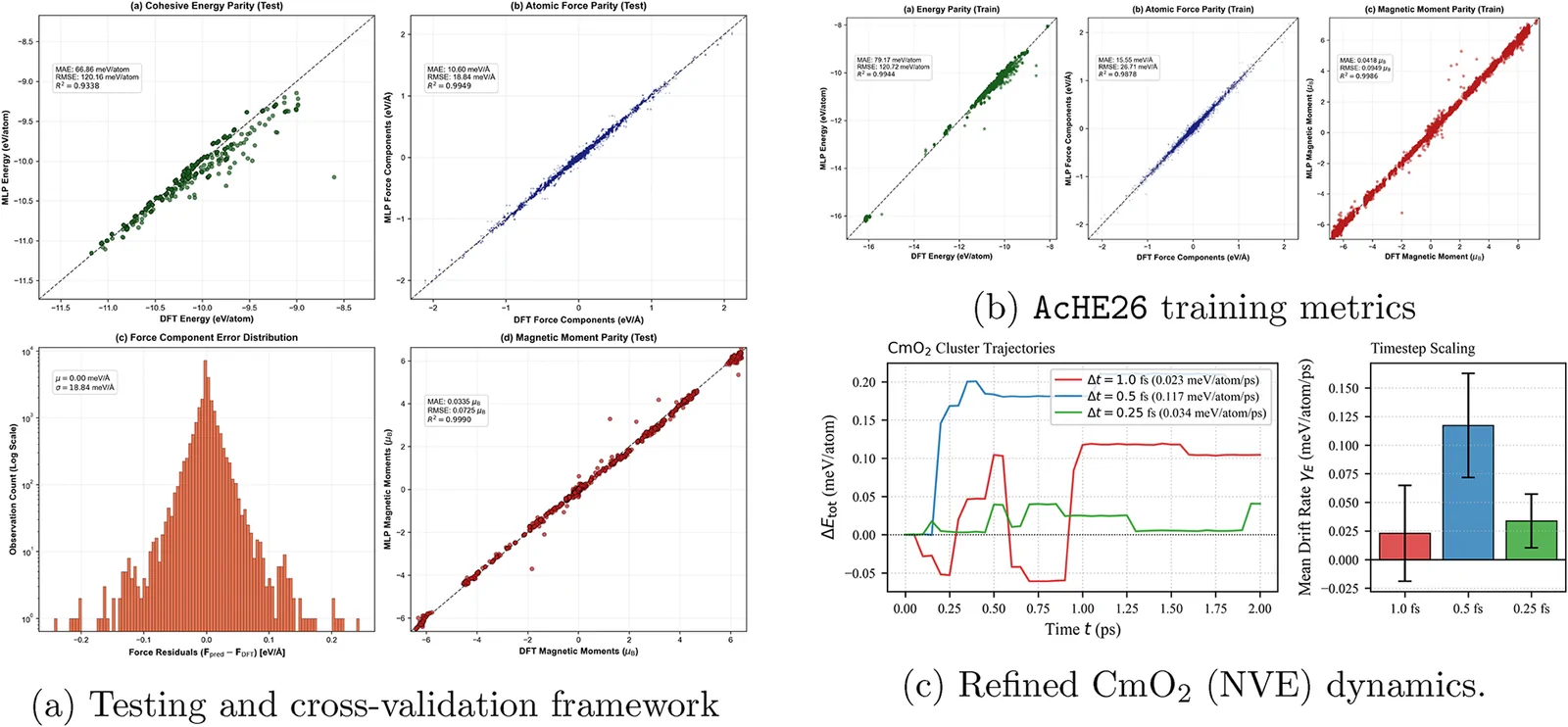
(a) Testing metrics on AcHE26 dataset. (b) Model optimization convergence after 100th epoch. (c) The resultant near-zero tracking stability during CmO_2_ NVE simulations.

### 4.8 Finite-Temperature Phase Stability via Langevin Molecular Dynamics

To investigate the finite-temperature dynamic stability, local coordination resilience, and structural integrity of the relaxed actinide binary oxide configurations (AmO_2_, and CmO). The crystallographic coordinates were anchored using verified experimental structures from the Crystallography Open Database (COD).^24,25^ We performed high-temperature simulations utilizing a Langevin thermostatting framework. The systems were instantaneously heated to a target production temperature of 1400 K. The classical equations of motion were integrated using a discrete time step of Δ*t* = 1.0 fs for a total production trajectory of 40.0 ps total simulation time.

Initial Configuration (*t* = 0) magnetic moments **μ** ^(0)^ are assigned based on a standard domain-specific initial guess of oxidation states or standard high-spin/low-spin state assumptions. During the MD simulations, magnetic moments propagate auto-regressively step-by-step **μ** ^(*t*)^ = **μ̂**^(*t*–1)^. At timestep *t*, the input interaction channels are constructed using the predicted magnetic moments **μ̂**^(*t*–1)^ output by the model at the immediately preceding step. These updated moments, combined with updated atomic positions **R** ^(*t*)^, are used to reconstruct the distance-weighted interaction matrices *M* ^(*t*)^. The network then evaluates the updated energy *E* ^(*t*)^, forces **F** ^(*t*)^, and predicts updated moments **μ̂**^(*t*)^ for the subsequent step.

The structural variations and thermal displacements induced at 1400 K were evaluated by calculating the partial Pair Radial Distribution Functions (*g*(*r*)), as summarized in Figure 11. For the CmO matrix (Figure 11d), a correlation peak centered near ∼2.5 Å is consistent with persistence of a first Cm–O coordination shell, while the broadening of the secondary Cm–Cm and O–O peaks is consistent with thermal vibration.^25^ For the AmO_2_ matrix (Figure 11b), the Am–O peak at ∼2.3 Å and its separation from the longer-range Am–Am correlations are likewise consistent with retention of the expected fluorite coordination topology under these thermal conditions.^26–28^ A short-range feature in the O–O partial centered near ∼1.2 Å coincides closely with the equilibrium bond length of a gas-phase O_2_ molecule (∼1.21 Å), suggestive of transient molecular oxygen formation consistent with established reports of oxygen loss in bulk AmO_2_.^29–31^ Notably, all three partials additionally exhibit a spurious short-range correlation feature centered near 1.0–1.5 Å, appearing in the Cm–O/Am–O, O–O, and Cm–Cm/Am–Am channels alike, irrespective of the differing ionic radii and equilibrium bond lengths expected for each pair type. Because the KEN architecture used here was trained exclusively on the HE26 dataset—which comprises near-equilibrium relaxed crystal structures and does not explicitly sample short-range, high-force configurations, this region falls entirely outside the model’s validated training domain. We therefore attribute this feature to a failure of the surrogate potential to reproduce the correct short-range repulsive wall in an under-sampled region of configuration space, rather than to architectural limitations. As this artifact directly affects the short-range portion of *g*(*r*) that would otherwise be used to benchmark coordination geometry, a quantitative comparison of these trajectories against *ab initio* molecular dynamics (AIMD) is not presently meaningful: any apparent agreement or discrepancy with a DFT reference could reflect this extrapolation error as readily as genuine finite-temperature physics. We therefore defer quantitative AIMD benchmarking of these trajectories to a subsequent study in which the training dataset is explicitly augmented with close-contact/high-force configurations sampled from finite-temperature MD.

**Figure 11:**
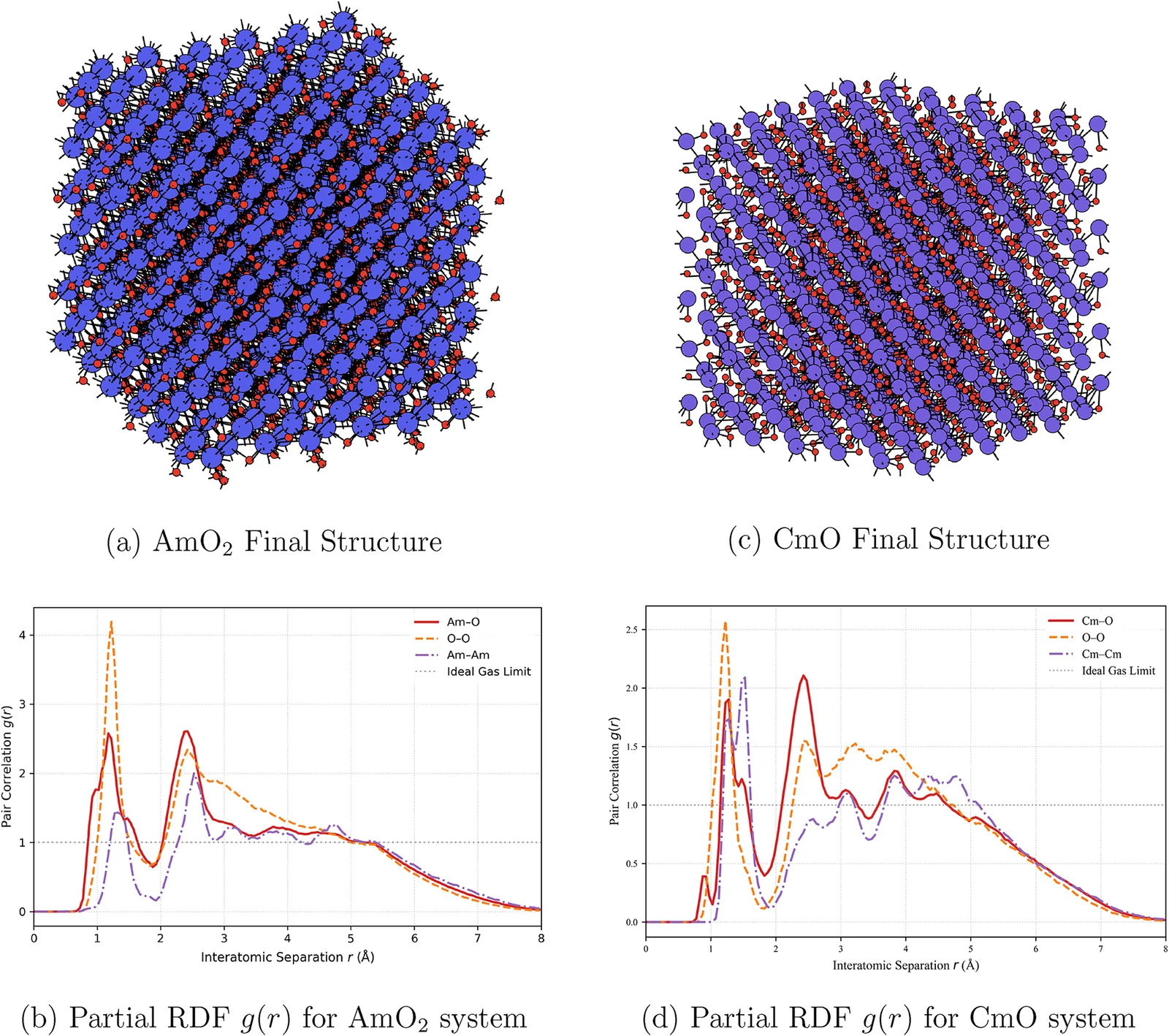
Analysis of local chemical environments extracted from 1400 K Langevin MD trajectories. (a) AmO_2_ fluorite supercell (6 × 6 × 6) at 4.0 ps timestamp. (b) Partial Pair Radial Distribution Function *g*(*r*) for AmO_2_ supercell. (c) Cubic supercell CmO (6 × 6 × 6) at 4.0 ps timestamp. (d) Partial Pair Radial Distribution Function *g*(*r*) for CmO supercell.

## 5 Conclusion

We report KEN, a multimodal architecture that integrates localized electronic spin degrees of freedom via a 2D CNN feature extractor with an *E*(3)-equivariant message-passing graph neural network. This hybrid design enables the model to efficiently learn complex potential energy surfaces in strongly correlated actinide systems without requiring constrained electronic or spin sampling. KEN achieves generalization and performance on par with MACE across the HE26 dataset while demonstrating enhanced architectural flexibility. We established the key role of our architecture in capturing localized magnetic couplings within chemically homogeneous sub-lattices, a capability that is crucial for actinide-bearing systems. Furthermore, KENJI demonstrates competitive performance against CHGNet for magnetic moment predictions. Crucially, while CHGNet is restricted to scalar magnitudes (|μ|), KEN natively predicts vector spin orderings. Because publicly available datasets remain skewed either chemically or magnetically, a dedicated follow-up study will be required to perform a fully unified benchmark of KEN against emerging magnetic MLIPs.

Bulk equation-of-state (EOS) calculations using the third-order Birch–Murnaghan formulation across the HE26 test set yield an equilibrium lattice parameter error of RMSE_*a*_0__ = 0. 5165 Å near equilibrium geometries. A small subset of americium-based molecular coordination complexes drives the volume and lattice parameter residuals higher, which is attributable to the unsuitability of isotropic Birch–Murnaghan strain models for soft organometallic packing modes. Because KENJI was trained without an explicit virial stress tensor loss (σ_αβ_), evaluating second derivatives of the energy under isotropic strain ( $B_{0}=V_{0}{\frac{d^{2}E}{{dV}^{2}}|}_{V_{0}}$ ) introduces expected variations relative to reference DFT+*U* bulk moduli (SI section 1).

Furthermore, finite-temperature simulations confirm the mathematical closure of the analytical automatic differentiation force graph. Under microcanonical (NVE) dynamics at 300 K, the model exhibits strong total energy conservation, demonstrating that dynamic gradient re-anchoring eliminates non-physical ghost forces across periodic boundaries. High-temperature (1400 K) Langevin molecular dynamics trajectories validate the structural resilience and mechanical stability of actinide oxide lattices over picosecond time scales. Emergent features in the oxygen partial radial distribution functions align conceptually with experimental reports of O_2_ gas release in bulk AmO_2_ under extreme thermal conditions. However, KENJI fails to reproduce the steep short-range repulsive wall in under-sampled regions of configuration space—a consequence of training on the near-equilibrium configurations of the HE26 dataset. This limitation underscores the need for chemically and magnetically balanced datasets tailored for universal MLIPs (uMLIPs).

Together, these results demonstrate the robustness of the KEN architecture and establish a viable pathway toward data-efficient, highly transferable interatomic potentials for large-scale simulations of microstructural evolution and thermal transport in nuclear fuels and strongly correlated functional materials.

## Supplementary Material



## Data Availability

The KENJIs utilized in this study, along with the hands-on scripts utilized in the present analysis, have been deposited in the GitHub repository and are accessible at https://github.com/gautam17phd/KEN-models.git.

## Notes

Additional hyperparameters, architecture settings, loss function definitions, and dynamic scaling benchmarks (Tables S1 and S2), and extended equation of state (EOS) details for bulk AmO_2_ and CmO systems (Figure S1) (PDF)

## References

[1] Batzner S., Musaelian A., Sun L., Geiger M., Mailoa J. P., Kornbluth M., Molinari N., Smidt T. E., Kozinsky B. (2022). E­(3)-equivariant
graph neural networks for data-efficient and accurate interatomic
potentials. Nat. Commun..

[2] Musaelian A., Batzner S., Johansson A., Sun L., Owen C. J., Kornbluth M., Kozinsky B. (2023). Learning local equivariant
representations
for large-scale atomistic dynamics. Nat. Commun..

[3] Drautz R. (2019). Atomic cluster
expansion for accurate and transferable interatomic potentials. Phys. Rev. B.

[4] Bochkarev A., Lysogorskiy Y., Menon S., Qamar M., Mrovec M., Drautz R. (2022). Efficient
parametrization of the atomic cluster expansion. Phys. Rev. Mater..

[5] Lysogorskiy Y., Oord C. v. d., Bochkarev A., Menon S., Rinaldi M., Hammerschmidt T., Mrovec M., Thompson A., Csányi G., Ortner C., Drautz R. (2021). Performant implementation of the
atomic cluster expansion (PACE) and application to copper and silicon. npj Comput. Mater..

[6] Batatia, I. ; Kovács, D. P. ; Simm, G. N. C. ; Ortner, C. ; Csányi, G. MACE: Higher Order Equivariant Message Passing Neural Networks for Fast and Accurate Force Fields 2022 https://arxiv.org/abs/2206.07697.

[7] Behler J., Parrinello M. (2007). Generalized
Neural-Network Representation of High-Dimensional
Potential-Energy Surfaces. Phys. Rev. Lett..

[8] Chapman J. B. J., Ma P.-W. (2022). A machine-learned spin-lattice potential for dynamic
simulations of defective magnetic iron. Sci.
Rep..

[9] Barry M. C., Wise K. E., Kalidindi S. R., Kumar S. (2020). Voxelized Atomic Structure
Potentials: Predicting Atomic Forces with the Accuracy of Quantum
Mechanics Using Convolutional Neural Networks. J. Phys. Chem. Lett..

[10] Novikov I., Grabowski B., Körmann F., Shapeev A. (2022). Magnetic Moment Tensor
Potentials for collinear spin-polarized materials reproduce different
magnetic states of bcc Fe. npj Comput. Mater..

[11] Rinaldi M., Mrovec M., Bochkarev A., Lysogorskiy Y., Drautz R. (2024). Non-collinear magnetic atomic cluster expansion for
iron. npj Comput. Mater..

[12] Khazieva E. O., Chtchelkatchev N. M., Katkov N. N., Ryltsev R. E. (2025). Accuracy and limitations
of machine-learned interatomic potentials for magnetic systems: A
case study on Fe-Cr-C. Phys. Rev. E.

[13] Kostiuchenko T. S., Shapeev A. V., Novikov I. S. (2024). Interatomic Interaction Models for
Magnetic Materials: Recent Advances. Chin. Phys.
Lett..

[14] Lukens W. W., Speldrich M., Yang P., Duignan T. J., Autschbach J., Kögerler P. (2016). The roles of 4f- and 5f-orbitals in bonding: a magnetochemical,
crystal field, density functional theory, and multi-reference wavefunction
study. Dalton Trans..

[15] Pepper M., Bursten B. E. (1991). The electronic structure
of actinide-containing molecules:
a challenge to applied quantum chemistry. Chem.
Rev..

[16] Vitova T., Pidchenko I., Fellhauer D., Bagus P. S., Joly Y., Pruessmann T., Bahl S., Gonzalez-Robles E., Rothe J., Altmaier M., Denecke M. A., Geckeis H. (2017). The role of
the 5f valence orbitals of early actinides in chemical bonding. Nat. Commun..

[17] Alzate-Vargas L., Subedi K. N., Lubbers N., Cooper M. W. D., Tutchton R. M., Gibson T., Messerly R. A. (2025). Toward
machine learning interatomic
potentials for modeling uranium mononitride. Mach. Learn.:Sci. Technol..

[18] Nguyen M.-T., Rousseau R., Paviet P. D., Glezakou V.-A. (2021). Actinide
Molten
Salts: A Machine-Learning Potential Molecular Dynamics Study. ACS Appl. Mater. Interfaces.

[19] Klawohn S., Darby J. P., Kermode J. R., Csányi G., Caro M. A., Bartók A. P. (2023). Gaussian
approximation potentials:
Theory, software implementation and application examples. J. Chem. Phys..

[20] Kuroda, N. ; Ishihara, K. ; Shiota, T. ; Mizukami, W. Expanding Universal Machine Learning Interatomic Potentials to 97 Elements Towards Nuclear Applications 2026 https://arxiv.org/abs/2603.03223.

[21] Heisenberg W. (1928). Zur Theorie
des Ferromagnetismus. Z. Phys..

[22] ishihara, k. HE26 2026 https://figshare.com/articles/dataset/HE26/31429325.

[23] Birch F. (1947). Finite Elastic
Strain of Cubic Crystals. Phys. Rev..

[24] Wyckoff, R. W. G. Crystal Structures, 2nd ed.; Interscience Publishers: New York, NY, 1963; Vol. 1, pp 239–444.

[25] Seleznev A. G., Radchenko V. M., Shushakov V. D., Ryabinin M. A., Droznik R. R., Lebedeva L. S., Vasilyev V. Y. (1990). Preparation of rare metallic actinides
(248Cm, 249Bk, 249Cf) and investigation of their crystal structure. J. Radioanal. Nucl. Chem. Artic..

[26] Sobolev V. (2009). Thermophysical
properties of NpO_2_, AmO_2_ and CmO_2_. J. Nucl. Mater..

[27] Talla
Noutack M. S., Geneste G., Jomard G., Freyss M. (2019). First-principles
investigation of the bulk properties of americium dioxide and sesquioxides. Phys. Rev. Mater..

[28] Lu Y., Yang Y., Zheng F., Wang B.-T., Zhang P. (2013). Electronic,
mechanical, and thermodynamic properties of americium dioxide. J. Nucl. Mater..

[29] Silva, R. J. ; Bidoglio, G. ; Rand, M. H. ; Robouch, P. B. ; Wanner, H. ; Neck, I. Chemical Thermodynamics of Americium; OECD Nuclear Energy Agency; Elsevier: Amsterdam, 1995.

[30] Prieur D., Martin P. M., Jankowiak A., Gavilan E., Scheinost A. C., Herlet N., Dehaudt P., Blanchart P. (2011). Local Structure
and Charge Distribution in Mixed Uranium-Americium Oxides: Effects
of Oxygen Potential and Am Content. Inorg. Chem..

[31] Gotcu-Freis, P. ; Konings, R. J. M. Oxygen potential measurements of americium oxides versus temperature. Technical Report INIS-XA-C36GX, 1995.

