# Supplementary material for: Convolutional Kernel-Embedded E(3)-Equivariant Networks (KEN): Overcoming Architectural Rigidity in Machine Learning Interatomic Potentials
Source: J Chem Theory Comput. 2026 Oct 2;22(21):11303–18. doi: 10.1021/acs.jctc.6c01109 (PMC13757765; doi:10.1021/acs.jctc.6c01109)
Supplement: Supplementary file 1 [file ct6c01109_si_001.pdf]

SUPPORTING INFORMATION

Convolutional Kernel-Embedded $\mathbb{E}(3)$-Equivariant Networks (KEN: Overcoming Architectural Rigidity in Machine Learning Interatomic Potentials

Jha Gautam*

Hylleraas Centre for Quantum Molecular Sciences, Department of Chemistry, University of Oslo, Postboks 1033 Blindern, Oslo, 0315, Norway

E-mail: gautam.jha@kjemi.uio.no

Reproducible Hyperparameters

Table S1: **Complete Computational, Architectural, and Training Parameters.**

Category	Parameter Value / Setting
**Data Splitting**	Train / Test Ratio: 95% / 5% (`official HE26` split)
**Model Architecture**	Equivariant spherical harmonics resolution: $l_{\max} = 2$<br>Invariant scalar channels ($\mathbf{h}\|_{0e}$): 128<br>Interaction cutoff radius (r_{cut}): 5.0 Å<br>2D CNN Channel Kernels: Two 3×3 convolutions followed by one 1×1 convolution, Channel depth = 32<br>Radial basis functions: 8 Bessel functions with polynomial cutoff ($p = 6$)
**Optimizer Settings**	Optimizer: AdamW ($\beta_1 = 0.9, \beta_2 = 0.999, \epsilon = 10^{-8}$)<br>Weight decay: 10^{-5}<br>Initial learning rate: 1×10^{-3}<br>Learning rate scheduler: `ReduceLROnPlateau`<br>Scheduler parameters: Factor = 0.5, Patience = 10 epochs, Min LR = 10^{-7}<br>Batch size: 16 structures per GPU (gradient accumulation step = 1)<br>Total epochs: 100 (with Early Stopping patience = 30 epochs)
**Loss Function Weights**	Energy loss weight (λ_E): 10.0 (MSE loss)<br>Force loss weight (λ_F): 1.0 (MSE loss)<br>Magnetic moment loss weight (λ_μ): 0.1 (MSE loss)
**Molecular Dynamics**	Integrator: Velocity Verlet (NVE) / Langevin thermostat (NVT)<br>Timestep (Δt): 0.5 fs<br>Time length (t_{tot}): 40.0 ps<br>Langevin friction coefficient (γ): 0.01 ps^{-1}<br>Domain initial guess ($t = 0$): (oxidation state, μ_B for magnetic cations)<br>Spin tracking ($t > 0$): Auto-regressive updates ($\boldsymbol{\mu}^{(t)} = \hat{\boldsymbol{\mu}}^{(t-1)}$)

Scaling Statistics

Table S2: Computational scaling, memory footprint, and energy-per-atom invariance benchmarks for KEN across varying supercell dimensions.

**Atoms (N)**	$E_{\textbf{total}}$ **(eV)**	E**/atom (eV)**	**Time / Step (ms)**	**Peak RSS (MB)**
96	−1057.0981	−11.01143932	519.40	748.2
324	−3567.8140	−11.01177120	2301.81	1643.4
768	−8457.1016	−11.01185131	6264.62	3377.7
1500	−16517.8203	−11.01187992	13953.57	6302.2
2592	−28542.8242	−11.01189232	20432.94	8979.2

Bulk Equation of State and Mechanical Response

To evaluate the smooth structural transferability and mechanical robustness of the trained potential under uniform lattice strains, we calculated the static total energy response of fluorite $2 \times 2 \times 2$ AmO_2 and cubic $2 \times 2 \times 2$ CmO across a volumetric deformation range of $\pm 10\%$. The crystallographic coordinates were anchored natively using verified experimental structures from the Crystallography Open Database (COD).[1,2] The resulting energy–volume (E–V) configurations were fitted to the third-order Birch–Murnaghan equation of state (EOS).[3]

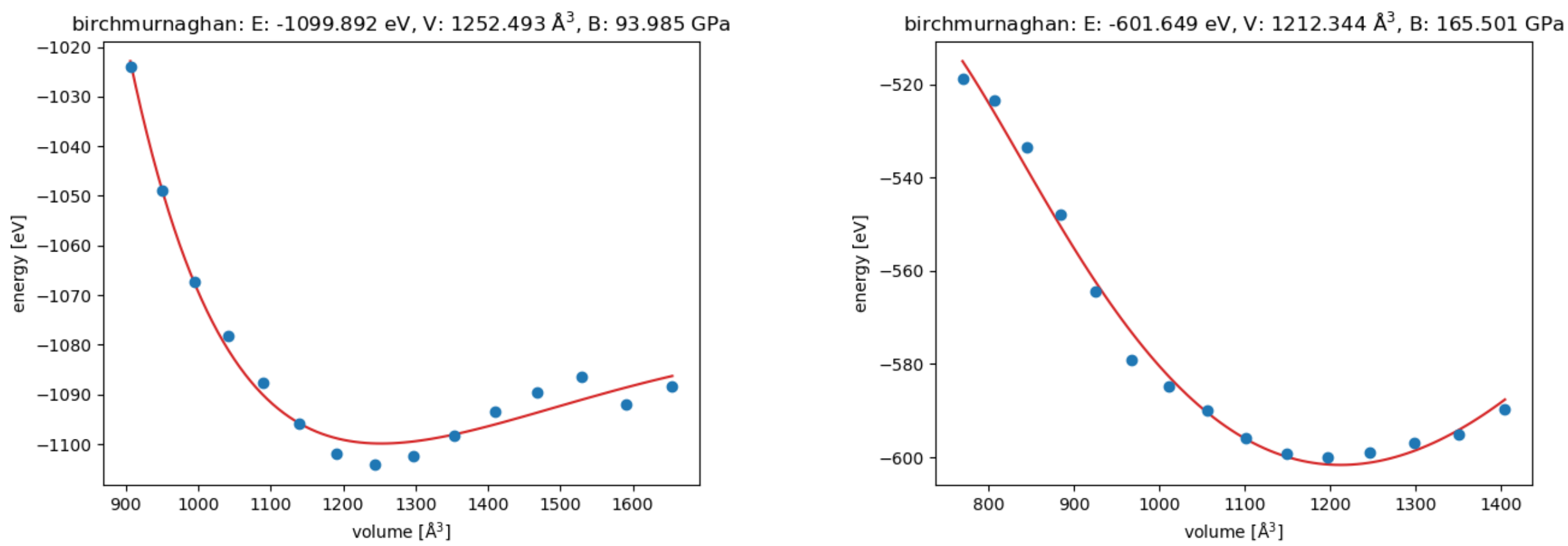

(A) AmO_2 Equation of State (B) CmO Equation of State

Figure S1: Calculated third-order Birch–Murnaghan equation of state curve for bulk (A) AmO_2 and (B) CmO. The smooth, continuous parabolic trajectory demonstrates the capacity of the KEN MLIP to maintain physical energy surfaces across compressed and expanded cell regions without introducing unphysical coordinate noise.

As shown in Figure S1, the KEN MLIP traces a smooth, numerically stable potential-energy curve over both compressed and expanded cell volumes without introducing unphysical coordinate noise. Fitting a third-order Birch–Murnaghan equation of state to this profile locates the equilibrium volume at 1252.493 Å^3 for AmO_2 and 1212.344 Å^3 for CmO, corresponding to lattice parameters of 5.389 Å and 5.331 Å, with 0.24% and 4.7% deviation from the COD cell parameters of 5.376 Å[1] and 5.091 Å,[2] respectively. The lattice parameter de-

viation for AmO_2 is remarkably small, whereas for CmO it is relatively large, which can be attributed to the fact that CmO is not a stable phase among curium oxides, meaning COD database values might not be precise. Overall, the EOS results demonstrate that the learned potential energy surface is well-behaved around equilibrium and structurally transferable under uniform volumetric deformation.

By utilizing an explicit seven-argument serialization trace that preserves the analytical derivative chain rule across cell translations ($\vec{r}_{ij} = \Delta\vec{R} + \mathrm{cell\_offsets}$), the model yields a bulk modulus of $B = 93.985\,\mathrm{GPa}$ for AmO_2 and $B = 165.501\,\mathrm{GPa}$ for CmO. The value for AmO_2 is significantly lower than the DFT+U range of 187–216 GPa reported in Refs. 4–6, whereas the value for CmO is quite close, indicating that the learned elastic curvature of the energy–volume surface is under-stiffened. A plausible contributing factor is that no stress information was included during training, meaning that the second derivatives of the energy with respect to homogeneous strain were only indirectly constrained through energies and forces. Despite this discrepancy in the absolute bulk modulus, the energy–volume curve exhibits a smooth basin curvature and a stable response under finite compression, indicating a well-behaved potential energy surface around equilibrium. In particular, the integrated 2D electronic matrix descriptors enable the model to consistently capture local bonding stiffness and qualitative compression trends.

References

(1) Wyckoff, R. W. G. *Crystal Structures*, 2nd ed.; Interscience Publishers: New York, NY, 1963; Vol. 1; pp 239–444.

(2) Seleznev, A. G.; Radchenko, V. M.; Shushakov, V. D.; Ryabinin, M. A.; Droznik, R. R.; Lebedeva, L. S.; Vasilyev, V. Y. Preparation of rare metallic actinides (248Cm, 249Bk, 249Cf) and investigation of their crystal structure. *Journal of Radioanalytical and Nuclear Chemistry Articles* **1990**, *143*, 253–259.

(3) Birch, F. Finite Elastic Strain of Cubic Crystals. *Physical Review* **1947**, *71*, 809–824.

(4) Talla Noutack, M. S.; Geneste, G.; Jomard, G.; Freyss, M. First-principles investigation of the bulk properties of americium dioxide and sesquioxides. *Physical Review Materials* **2019**, *3*.

(5) Lu, Y.; Yang, Y.; Zheng, F.; Wang, B.-T.; Zhang, P. Electronic, mechanical, and thermodynamic properties of americium dioxide. *Journal of Nuclear Materials* **2013**, *441*, 411–420.

(6) Sobolev, V. Thermophysical properties of NpO_2, AmO_2 and CmO_2. *Journal of Nuclear Materials* **2009**, *389*, 45–51.
